# Bio-fabrication of silver nanoparticles by phycocyanin, characterization, *in vitro* anticancer activity against breast cancer cell line and *in vivo* cytotxicity

**DOI:** 10.1038/s41598-017-11121-3

**Published:** 2017-09-07

**Authors:** Noura El-Ahmady El-Naggar, Mervat H. Hussein, Asmaa Atallah El-Sawah

**Affiliations:** 1 Department of Bioprocess Development, Genetic Engineering and Biotechnology Research Institute, City of Scientific Research and Technological Applications, Alexandria, Egypt; 2 Botany Department, Faculty of Science, Mansoura University, Mansoura, Egypt

**Keywords:** Nanofabrication and nanopatterning, Nanoparticles

## Abstract

In recent decades, researchers were attracted towards cyanobacterial components which are potential low-cost biological reagents for silver nanoparticle biosynthesis. This article describes the biological synthesis of silver nanoparticles using a proteinaceous pigment phycocyanin extracted from *Nostoc linckia* as reducing agent. The synthesized silver nanoparticles have a surface plasmon resonance band centered at 425 nm. Face-centered central composite design used for optimization of silver nanoparticles (AgNPs) biosynthesis using phycocyanin. The maximum AgNPs biosynthesis obtained using the optimized four variables, initial pH level (10), AgNO_3_ concentration (5 mM), phycocyanin pigment concentration (1 mg/mL) and incubation period (24 h) was 1100.025 µg/mL. The TEM analysis of AgNPs showed spherical nanoparticles with mean size between 9.39 to 25.89 nm. FTIR spectra showed major peaks of proteins involved in AgNPs biosynthesis by identifying different functional groups involved in effective capping of AgNPs. The biosynthesized AgNPs significantly inhibited the growth of medically important pathogenic Gram-positive (*Staphylococcus aureus*), Gram-negative bacteria (*Pseudomonas aeruginosa, E. coli* and *Klebsiella pneumonia*). The synthesized AgNPs exhibited effective cytotoxic activity against MCF-7 and the inhibitory concentration (IC_50_) was recorded at 27.79 ± 2.3 µg/mL. The *in vivo* studies clearly indicated that AgNPs has a capacity to inhibit the growth of tumor in Ehrlich ascites carcinoma bearing mice.

## Introduction

The great interest in the synthesis of metal and metal oxide based nanoparticles through chemical, physical and biological routes is due to their extraordinary abilities to function as catalysts and help in numerous processes of physics, chemistry, biology, medicine, engineering, and informatics^1^. Physical and chemical methods are currently widely used to synthesize metal nanoparticles. The conventional production methods are usually expensive, labor-intensive and produce hazard byproducts which are harmful to the environment and living organisms^2^. Subsequently, there is an intensive need for using ecofriendly, safe, reliable and clean methods for the preparation of nanoparticles^3^. Various biological routes are considered safe, non toxic and providing a more environmentally sound synthesis of nanoparticles, including the use of plant extracts^4^, *Streptomyces*^5^, bacteria^6^, fungi^7^ and algae^8^. Although cyanobacteria is consider the best biological system for nanoparticles synthesis intracellulary and extracellulary, there are few reports about biological synthesis of noble metal nanoparticles using it^9^. Extracellulary, *Sargassum wightii* and *Kappaphycus alvarezii* have been used for synthesis of metal nanoparticles by Singaravelu *et al*.^10^ and Rajasulochana *et al*.^11^, respectively. Intracellulary, Senapati *et al*.^12^, reported the production of gold nanoparticles using *Tetraselmis kochinensis. Spirulina platensis* were also used for the synthesis of AgNPs^13^. The reduction of Ag^+^ ions occurs extracellular through reductase enzymes and electron shuttle quinones^14^. Silver ions are reduced intracellulary by electrons produced by the organisms to avert destruction in the presence of enzymes such as NADH-dependent reductases^15^. Cyanobacterial extracts like phycocyanin and polysaccharides can extracellulary reduce silver ions. Patel *et al*.^16^ reported that the phycocyanin extracted from *Limnothix* sp. 37-2-1 formed spherical and elongated AgNPs and spherical shape AgNPs formed from the phycocyanin extracted from *Spirulina* sp. Silver nanoparticles (AgNPs) via various metal nanoparticles have obtained significant consideration, because they are effective antimicrobial agents that shows low toxicity also have various *in vitro* and *in vivo* applications^17^. The high efficient antibacterial activity of AgNPs is due to the large surface area that comes in contact with the microbial cells and they have a higher percentage of interaction than larger particles of the same parent material^18^. Recently AgNPs are combined with medical supplements, catheters, wound dressings and implants routinely for inhibition pathogen growth and also incorporate to cosmetics as an antiseptic and used in medical textiles to eliminate microbes from the clinical environment^19^. AgNPs have a cytotoxic effect on cancer cells that AgNPs toxicity depends on their size, surface functionalization and concentration^20^.

In this study, we have investigated the biosynthesis of AgNPs through phycocyanin extracted from *Nostoc linckia* using face-centered central composite design which is capable to find optimum concentration of AgNO_3_, pH, incubation period and concentration of phycocyanin affecting the biosynthesis of AgNPs. Also, we have investigated the cytotoxic activity of AgNPs against mammary gland breast cancer (MCF-7) cell line *in vitro*, *in vivo* cytotxicity, the hemolytic and antibacterial activities.

## Results and Discussion

### Spectral characteristics of phycocyanin

**Figure 1:**
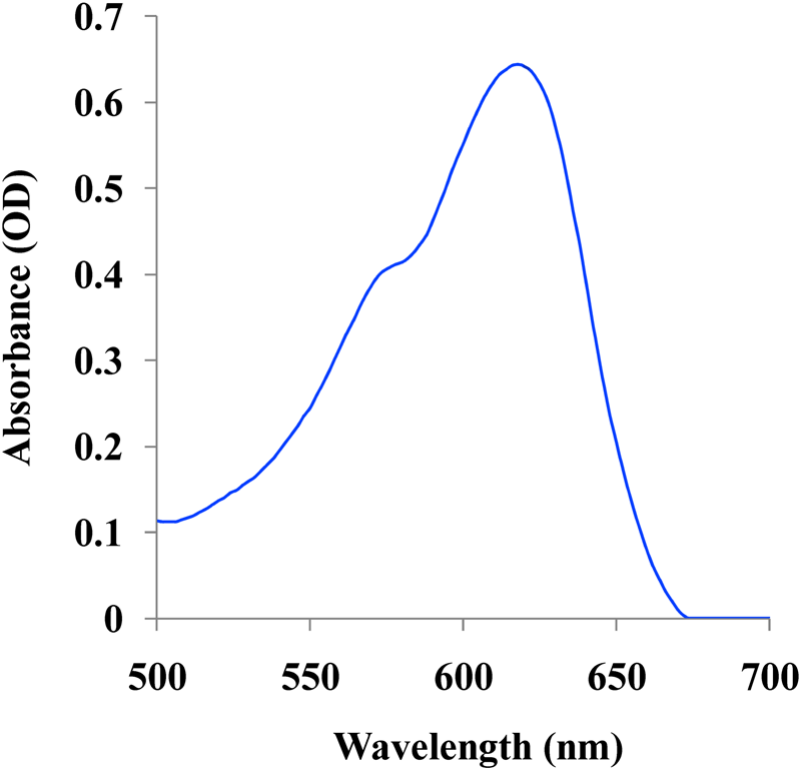
UV–Vis absorption spectrum of phycocyanin pigment with λ_A max_ = 614 nm.

The photosynthesis in cyanobacteria can take over broad region (450 to 650 nm) of solar spectrum due to the presence of brilliantly colored protein-based pigment called phycobiliprotein which is the family of the colored water-soluble pigment proteins^21^. Phycobiliproteins are classified according to their spectral properties. The maximum absorbance wavelength of phycocyanin (PC) λ_A max_ = 610–620 nm, phycoerythrin (PE) λ_A max_ = 540–570 nm, and allophycocyanin (APC) λ_A max_ = 650–655 nm which are the majorly found phycobiliproteins^22^. In this research, phycocyanin extracted from *Nostoc linckia* has λ_A max_ = 614 nm as showed in Fig. 1.

### Evaluation of silver nanoparticles biosynthesis by phycocyanin extracted from cyanobacterium *Nostoc linckia*

**Figure 2:**
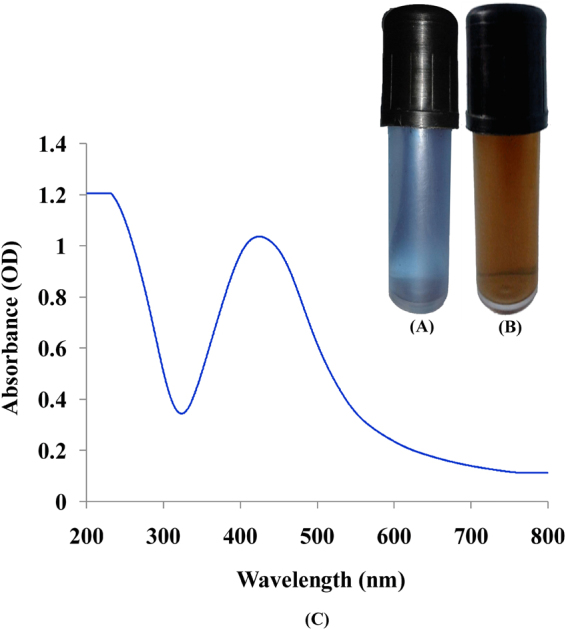
(**A**) Phycocyanin pigment (**B**) visible observation of AgNPs biosynthesis by phycocyanin pigment after exposure to AgNO_3_ solution (5 mM). (**C**) UV–Vis absorption spectra of silver nanoparticles synthesized by phycocyanin pigment.

Silver nanoparticles biosynthesis was carried out by using 19 ml of phycocyanin extracted from cyanobacterium *Nostoc linckia* treated with 1 mL (5 mM) aqueous AgNO_3._ The reaction mixture was kept in light. The phycocyanin colored reaction mixture converted to brown color indicating the formation of AgNPs (Fig. 2A,B). Conversely, no change in color observed in aqueous AgNO_3_ incubated without phycocyanin under the same conditions. Formation of color is depending on the excitation of surface Plasmon vibrations of silver nanoparticles^23^. Light-assisted methods for nanoparticles synthesis are established on reducing the cation of the metal M^n+^ to M^0^ either by direct or indirect (photosensitized) photolysis, which largely developed since 18^th^ century^24^. In this study, the phycocyanin chromophores which responsible for conformational change of the phycocyanin blue color initiate the photosynthesis in *Nostoc linckia* by the absorption of light. Chromophores can excite the molecules from the ground state to an electronic excited state after absorbing light^25^. Thus under illumination condition, the electrons which jump between energy levels can also reduce AgNO_3_ in the medium to form silver nanoparticles.

### UV-Visible spectral analysis

This investigation focused on the formation of AgNPs was proven using scanning UV-visible spectrophotometry in the range of 200–900 nm, over the sequence of time interval. The specific surface plasmon resonance (SPR) spectrum of silver nanoparticles produced by phycocyanin pigment revealed an absorption peak at 425 nm (Fig. 2C), this result was compatible with the result of Yadav *et al*.^26^. AgNPs biosynthesis using algal and cyanobacterial cells were followed by change in UV–vis absorbance peak associated with surface plasmon resonance of the AgNO_3_ solution^16^. The plasmon absorption size and shape of silver nanoclusters are strongly depend on particle size, stabilizing molecules or surface adsorbed particles as well as the dielectric constant of the medium^17^. With increasing in size of silver nanoparticles in aqueous solution, surface plasmon resonance peak shifts to longer wavelengths.

### Response surface methodology for optimization of silver nanoparticles biosynthesis and model analyzing

Response surface methodology (RSM) is used to determine the optimum variables concentrations affecting silver nanoparticles biosynthesis. In this study, the face-centered central composite design (FCCD) was applied to design the experiments and evaluate the interactive effects of the four selected variables. The experiments were designed using the software, Design Expert® 7.0 for Windows.

Table 1 presents the face-cantered central composite design matrix of four independent variables X_1_ (initial medium pH), X_2_ (AgNO_3_ concentration), X_3_ (phycocyanin concentration) and X_4_ (incubation period) with their actual and coded levels. Thirty experiments were performed contain 18 factorial, 6 axial and 6 central points. The six central points are found in runs order 5, 14, 19, 21, 24 and 29. A maximum biosynthesis of silver nanoparticles value was (1100.025 µg/mL) which is the mean value of runs order 5, 14, 19, 21, 24 and 29. The maximum biosynthesis of silver nanoparticles was achieved under the experimental conditions of initial medium pH level (10), AgNO_3_ concentration (5 mM), phycocyanin pigment concentration (1 mg/mL) and incubation period (24 h). However, a minimum biosynthesis of silver nanoparticles values (330.046 µg/mL) was found in experiment number run order 10 under the conditions of initial pH level (12), AgNO_3_ concentration (1 mM), phycocyanin pigment concentration (2 mg/mL) and incubation period (2 h). The experimental and predicted values of yields of silver nanoparticles biosynthesis are also given in Table 2.

**Table 1:** Face-centered central composite design matrix of four process variables with actual factor levels corresponding to coded factor levels, mean experimental and predicted values of silver nanoparticles biosynthesis by phycocyanin pigment.

Std	Run	Type	Initial pH level (X_1_)		AgNO_3_ concentration (X_2_)		Phycocyanin pigment concentration (X_3_)		Incubation period (X_4_)		AgNPs (µg/mL)		Residuals
			Coded	Actual	Coded	Actual (mM)	Coded	Actual (mg/mL)	Coded	Actual (h)	Experimental	Predicted	
15	1	Factorial	−1	5	1	50	1	2	1	72	458.270	377.329	80.941
17	2	Axial	−1	5	0	5	0	1	0	24	933.229	973.112	−39.883
19	3	Axial	0	10	−1	1	0	1	0	24	467.375	478.667	−11.292
22	4	Axial	0	10	0	5	1	2	0	24	867.223	950.874	−83.651
30	5	Center	0	10	0	5	0	1	0	24	1100.025	1064.032	35.993
5	6	Factorial	−1	5	−1	1	1	2	−1	2	420.334	390.620	29.714
8	7	Factorial	1	12	1	50	1	2	−1	2	554.640	483.866	70.774
10	8	Factorial	1	12	−1	1	−1	0.5	1	72	540.212	596.125	−55.913
3	9	Factorial	−1	5	1	50	−1	0.5	−1	2	344.461	380.805	−36.344
6	10	Factorial	1	12	−1	1	1	2	−1	2	330.046	370.916	−40.870
13	11	Factorial	−1	5	−1	1	1	2	1	72	361.153	429.407	−68.254
4	12	Factorial	1	12	1	50	−1	0.5	−1	2	580.425	566.716	13.709
9	13	Factorial	−1	5	−1	1	−1	0.5	1	72	374.810	373.043	1.767
28	14	Center	0	10	0	5	0	1	0	24	1100.025	1064.032	35.993
20	15	Axial	0	10	1	50	0	1	0	24	471.168	531.862	−60.694
2	16	Factorial	1	12	−1	1	−1	0.5	−1	2	399.848	408.248	−8.400
24	17	Axial	0	10	0	5	0	1	1	72	1170.251	1167.922	2.329
23	18	Axial	0	10	0	5	0	1	−1	2	1031.108	1105.424	−74.316
26	19	Center	0	10	0	5	0	1	0	24	1100.025	1064.032	35.993
1	20	Factorial	−1	5	−1	1	−1	0.5	−1	2	330.061	285.698	44.363
29	21	Center	0	10	0	5	0	1	0	24	1100.025	1064.032	35.993
18	22	Axial	1	12	0	5	0	1	0	24	1074.379	1106.482	−32.103
12	23	Factorial	1	12	1	50	−1	0.5	1	72	695.751	652.924	42.827
25	24	Center	0	10	0	5	0	1	0	24	1100.025	1064.032	35.993
21	25	Axial	0	10	0	5	−1	0.5	0	24	975.781	964.116	11.665
16	26	Factorial	1	12	1	50	1	2	1	72	422.610	521.518	−98.908
14	27	Factorial	1	12	−1	1	1	2	1	72	619.120	510.235	108.885
7	28	Factorial	−1	5	1	50	1	2	−1	2	441.578	440.209	1.369
27	29	Center	0	10	0	5	0	1	0	24	1100.025	1064.032	35.993
11	30	Factorial	−1	5	1	50	−1	0.5	1	72	352.807	366.481	−13.674

**Table 2:** Regression coefficients of second order polynomial model for optimization of silver nanoparticles biosynthesis by phycocyanin pigment.

Factor	Coefficient estimate	*df*	Standard error	95% CI Low	95% CI High
Intercept	1064.03	1	22.11	1016.91	1111.15
X_1_	66.68	1	16.77	30.93	102.44
X_2_	26.60	1	16.77	−9.16	62.35
X_3_	−6.62	1	16.77	−42.38	29.13
X_4_	31.25	1	16.77	−4.51	67.00
X_1_ X_2_	15.84	1	17.79	−22.08	53.76
X_1_ X_3_	−35.56	1	17.79	−73.49	2.36
X_1_ X_4_	25.13	1	17.79	−12.79	63.06
X_2_ X_3_	−11.38	1	17.79	−49.30	26.54
X_2_ X_4_	−25.42	1	17.79	−63.34	12.51
X_3_ X_4_	−12.14	1	17.79	−50.06	25.78
X_1_^2^	−24.23	1	44.22	−118.48	70.01
X_2_^2^	−558.77	1	44.22	−653.01	−464.52
X_3_^2^	−106.54	1	44.22	−200.78	−12.29
X_4_^2^	72.64	1	44.22	−21.60	166.88

*Significant values, *df*: Degree of freedom.

### Multiple regression analysis and ANOVA

The relationship between a set of independent variables and silver nanoparticles biosynthesis is determined by a mathematical model called multiple-regression model using Design Expert 7.0 for Windows. Statistical analysis of the response was performed, summarized and presented in Tables 2 and 3 and Supplementary Table S1. In Table 2 the positive coefficients for X_1_, X_2_, X_4_, X_1_ X_2_, X_1_ X_4_, X_4_^2^ indicate that linear effect of X_1_, X_2_, X_4_ interaction effects for X_1_ X_2_, X_1_ X_4_ and the quadratic effect of X_4_^2^ increase silver nanoparticles biosynthesis, while other negative coefficients indicate decrease in silver nanoparticles biosynthesis.

**Table 3:** Regression statistics and analysis of variance (ANOVA) for face-centered central composite design experimental values of silver nanoparticles biosynthesis by phycocyanin pigment.

Source	Sum of Squares	*df*	Mean Square	*F-*value	*P-*value *P*rob > *F*	Confidence Level
Model	2798066.14	14	199861.87	39.46	<0.0001	99.99
X_1_	80043.74	1	80043.74	15.80	0.0012	99.88
X_2_	12733.47	1	12733.47	2.51	0.1337	86.63
X_3_	789.13	1	789.13	0.16	0.6986	30.14
X_4_	17577.06	1	17577.06	3.47	0.0822	91.78
X_1_ X_2_	4014.55	1	4014.55	0.79	0.3874	61.26
X_1_ X_3_	20236.20	1	20236.20	4.00	0.0641	93.59
X_1_ X_4_	10106.68	1	10106.68	2.00	0.1782	82.18
X_2_ X_3_	2071.80	1	2071.80	0.41	0.5321	46.79
X_2_ X_4_	10336.38	1	10336.38	2.04	0.1736	82.64
X_3_ X_4_	2357.83	1	2357.83	0.47	0.5055	49.45
X_1_^2^	1521.71	1	1521.71	0.30	0.5917	40.83
X_2_^2^	808935.93	1	808935.93	159.70	<0.0001	99.99
X_3_^2^	29407.04	1	29407.04	5.81	0.0293	97.07
X_4_^2^	13671.38	1	13671.38	2.70	0.1212	87.88
Residual	75978.06	15	5065.20			
Lack of Fit	75978.06	10	7597.81			
Pure Error	0	5	0			
Cor Total	2874044.20	29				
Std. Dev.	71.17	R-Squared	0.9736			
Mean	693.89	Adj R-Squared	0.9489			
C.V.%	10.26	Pred R-Squared	0.8250			
PRESS	503040.93	Adeq Precision	17.5305			

*Significant values, *df*: Degree of freedom, *F*: Fishers’s function, *P*: Level of significance, C.V: Coefficient of variation.

In order to evaluate the relationship between dependent and independent variables and to determine the maximum silver nanoparticles biosynthesis to the optimum levels of the four independent variables X_1_ (initial medium pH), X_2_ (AgNO_3_ concentration), X_3_ (phycocyanin concentration) and X_4_ (incubation period), a second-order polynomial mathematical model (Eq. 1) was proposed to calculate the optimum levels of these variables and defines predicted response (Y) in terms of the independent process variables:

1 $$\begin{array}{c}{\rm{Y}}=1064.03+66.68{{\rm{X}}}_{1}+26.60{{\rm{X}}}_{2}-6.62{{\rm{X}}}_{3}+31.25{{\rm{X}}}_{4}+15.84{{\rm{X}}}_{1}{{\rm{X}}}_{2}\\ \quad \quad -35.56{{\rm{X}}}_{1}{\rm{X}}3+25.13{{\rm{X}}}_{1}{{\rm{X}}}_{4}-11.38{{\rm{X}}}_{2}{{\rm{X}}}_{3}-25.42{{\rm{X}}}_{2}{{\rm{X}}}_{4}\\ \quad \quad -12.14{{\rm{X}}}_{3}{{\rm{X}}}_{4}-24.23{{\rm{X}}}_{1}^{2}-558.77{{\rm{X}}}_{2}^{2}-106.54{{\rm{X}}}_{3}^{2}+72.64{{\rm{X}}}_{4}^{2}\end{array}$$

where, Y is the predicted response (silver nanoparticles biosynthesis), X_1_ is initial medium pH, X_2_ is the AgNO_3_ concentration, X_3_ is the phycocyanin pigment concentration and X_4_ is the incubation period.

The coefficient of determination R^2^ is a widely accepted parameter for verification of the model adequacy^27^. The adequate of the model was also expressed by the coefficient of determination (R^2^) which was found to be 0.9736 (Table 3) indicating that 97.36% of variability in the silver nanoparticles biosynthesis could explained by the model and only 2.64% of the total variance could not be explained by the model. Box *et al*.^28^ suggested calculating the adjusted R^2^, because it can corrects the R^2^ value for the sample size and the numbers of terms of the model. The “Pred R-Squared” of 0.8250 is in reasonable agreement with the “Adj R-Squared” of 0.9489. This indicated a good adjustment between the observed and predicted values.

The analysis of variance (ANOVA) of the experimental design was calculated, and the sum of square, mean square, *F*-value, *P*-value and confidence level are shown in Table 3. The Model *F*-value of 39.45 implies that the model is significant where Model *P*-value (Prob > *F*) is very low which less than (0.0001). Also the probability values (*P* values) were used to check the significance of each of the coefficients, which, in turn, are necessary to understand the pattern of the mutual interactions between the test variables. The corresponding *P* values, along with the coefficient estimate, are given in Table 3. The smaller *P* values the more significant is the corresponding coefficient^29^. Some investigators have found that confidence levels greater than 70% are acceptable^30^. Variables at confidence levels greater than 90% (*P* < 0.1) were considered significant^31^. Thus, in the current experiment, variables evidencing *P*-values of less than 0.1 (confidence levels exceeding 90%) were considered to have significant effects on silver nanoparticles biosynthesis. In this study, the linear coefficient terms X_1_ (initial medium pH) and X_4_ (incubation period) affect significantly (*P* < 0.1) on the silver nanopaticles biosynthesis. Where the linear coefficients of X_3_ (initial medium pH) is significant (*P* value 0.0012), indicating that 99.88% of the model affected by the level of pH and the linear coefficients terms of X_4_ (incubation period) is significant (*P* value 0.0822), indicating that 91.78% of the model affected by the incubation period. The mutual interaction coefficient terms between X_1_ (initial medium pH) and X_3_ (phycocyanin concentration) with (*P* value 0.0641) also indicating that 93.95% of the model significantly affected by the interaction of these variables. The quadratic coefficient term of X_3_^2^ (phycocyanin concentration) showed (*P* value 0.0293) indicating that 97.07% of the model affected by the phycocyanin pigment concentration and X_2_ (AgNO_3_ concentration) showed (*P* value ˃ 0.0001) indicating that 99.99% of the model affected by AgNO_3_ concentration. However, the linear coefficient terms (X_2_ and X_3_), the interaction coefficient terms between (X_1_ X_2_, X_1_ X_4_, X_2_ X_3_, X_2_ X_4_ and X_3_ X_4_) and the quadric coefficient terms (X_1_^2^ and X_4_^2^) were found to be insignificant.

“Adeq Precision” measures the signal to noise ratio. A ratio greater than 4 is desirable. “Adeq Precision” ratio of 17.5286 indicates an adequate signal to noise ratio. Also in Table 3, the value of PRESS is 503061.47; standard deviation values is 71.17 and mean value is 693.89 with C.V. value equals 10.26, which was very low value, indicating that there is an accuracy and fitness in this experimental execution^28^. The fit summary results are presented in Supplementary Table S1.

**Figure 3:**
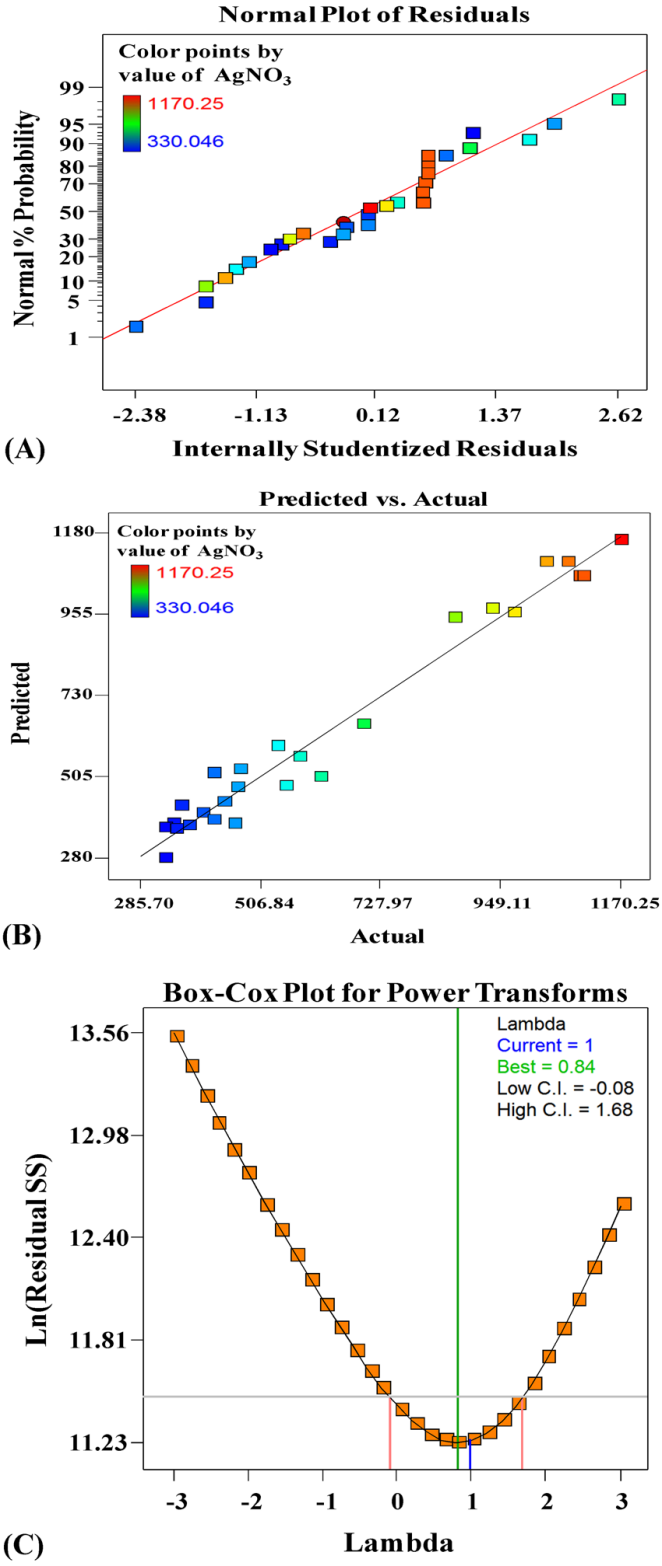
(**A**) The normal probability plot of the residuals. (**B**) Correlation between the experimented and predicted values for silver nanoparticles biosynthesis using phycocyanin pigment determined by the second-order polynomial equation. (**C**) Box-Cox plot of model transformations.

The normal probability plot is important a diagnostic tool that indicates whether the residuals follow a normal distribution, in which case the points will follow a straight line expect some scatter even with normal data. Figure 3A represents as the residuals from the fitted model were normally distributed along the silver nanoparticles biosynthesis straight line, indicating that the model had been validated. Figure 3B presents a plot of predicted versus experimental values of silver nanoparticles biosynthesis as a visual diagnostic plot indicated that, there is a close agreement between the experimental results and theoretical values predicted by the model equation, which confirms the adequacy of the model. As observed from Fig. 3C, the blue line indicates the current transformation (Lambda = 1) and the green line indicates the best lambda value (Lambda = 0.84), while the red lines indicate the minimum and maximum 95% confidence interval values (−0.08 and 1.68 respectively). Therefore, the model needs no transformation, as current value of confidence interval (λ = 1) is very close to model design value (best = 0.84) and the model is in the optimal zone since the blue line falls within the red lines. So that the model is well fit to the experimental data obtained and well satisfies the assumptions of the analysis of variance.

**Figure 4:**
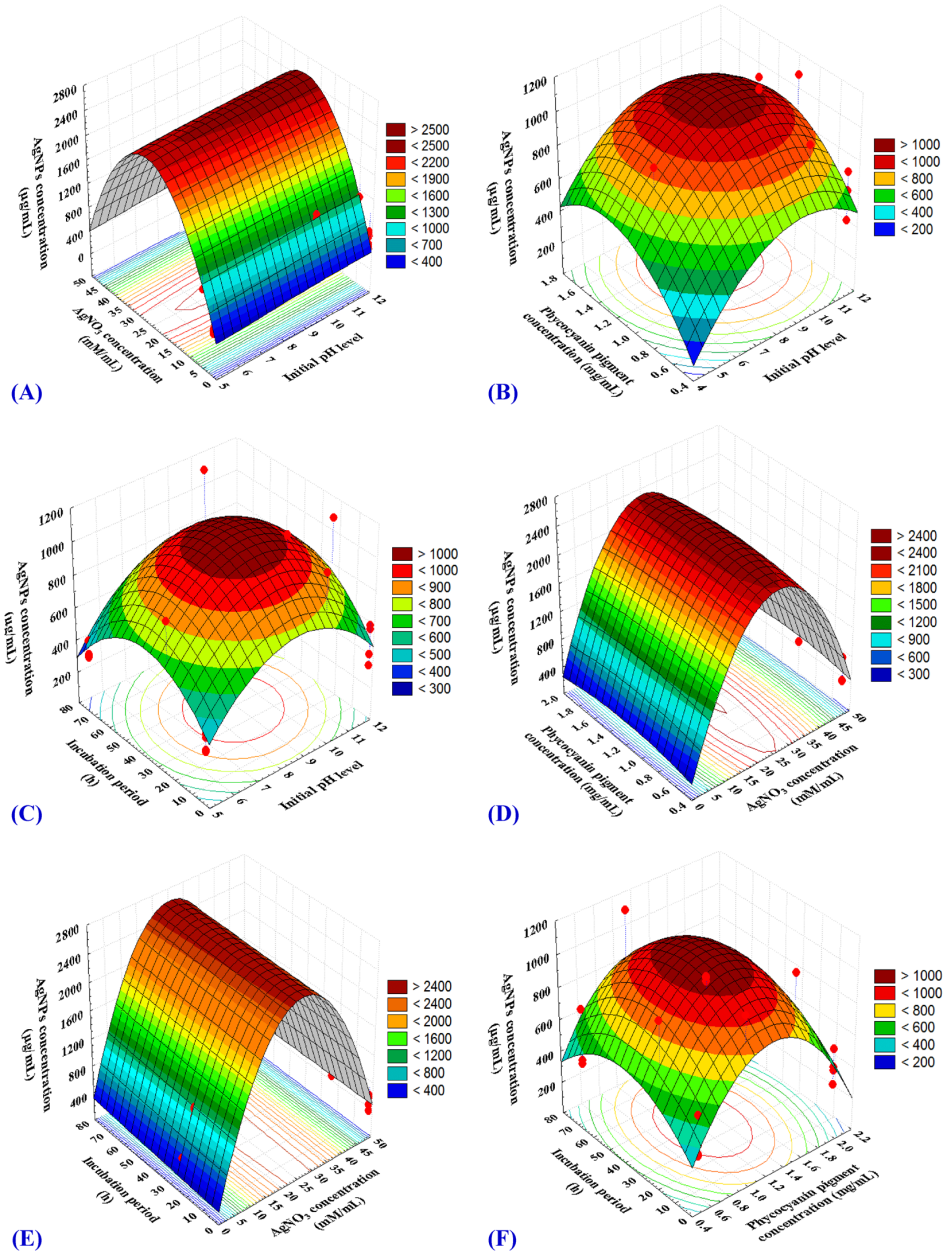
Three-dimensional response surface plots (**A**–**F**) showing the interactive effects of independent variables: initial pH level, AgNO_3_ concentration, phycocyanin pigment concentration and incubation period on biosynthesis of silver nanoparticles.

Three-dimensional graphs were generated for the pair-wise combination of the four factors, the silver nanoparticles biosynthesis on z-axis against two independent variables while keeping the other variables are held at zero level. Figure 4A represents the effect X_1_ (initial medium pH level) and X_2_ (AgNO_3_ concentration), while X_3_ (phycocyanin concentration) and X_4_ (incubation period) were held at their zero levels (1 mg/mL and 24 h; respectively). It showed that the maximum silver nanoparticles yield appeared at alkaline initial pH. The silver nanoparticles biosynthesis increases rapidly by increasing initial medium pH level with the increase of AgNO_3_ concentration until middle point, but further increase in the pH concentration led to the gradual decrease in the silver nanoparticles biosynthesis due to denaturation of the phycocyanin pigment.

Heydari and Rashidipour^32^ reported that the rate of AgNPs synthesis increases with increasing pH up to 9 pH then decrease. The physical factor (pH) affects on the size and the distribution of the nanoparticles. Some authers reported that the basic pH make a rapid growth rate^33^. Perfect yield and mono dispersity, size of AgNPs produced by casein milk protein were at pH > 7 ranged between 3 to 18 nm and in pH < 6 ranged between 60 to 80 nm^34^. By increasing the pH of the mixture, small spherical nanoparticles were formed under conditions of pH = 11using starch as capping agent^35^. Spherical size (7–20 nm) of AgNPs were observed in crude hot water soluble polysaccharide extracted from different marine algae with 0.1 mM of AgNO_3_ in pH = 10^36^. However, at higher pH (>11 pH), silver ions will aggregated and unstable AgNPs will appear^37^. However, face-centered cubic structure with spherical shape (5–25 nm) in size with filtered aqueous extract of *Caulerpa racemosa* marine algae as reducing agent^38^, crystal structure with spherical shape ranged between 28–41 nm in size using ethyl acetate extract of *Ulva fasciata* as reducing agent^39^ were obtained at neutral pH. Acidic medium also have been used in biosynthesis of AgNPs but in a little manner. Finally, alkaline pH is the perfect medium for AgNPs synthesis like in our research pH = 10 that was ratified by El-Rafie *et al*.^36^.

The reduction of Ag+ ions to Ag° nanoparticles was indicated by changing the color of any given solution mixture to yellowish–brown gradually increasing to deep brown color depending on both concentration of silver nitrate and the reducing agent. Authors use a wide range of AgNO_3_ concentration in the biosynthesis on AgNPs ranging from 1 mM to 10 mM. 1 mM of AgNO_3_ is the most common used concentration for AgNPs biosynthesis. Kathiraven *et al*.^38^ used 1 mM of silver nitrate with algal extracts for production AgNPs. El-Naggar and Abdelwahed^40^ and El-Naggar *et al*.^5^ used 1 mM AgNO_3_ in AgNPs biosynthesis by a nanofactory *Streptomyces viridochromogenes* and *Streptomyces* sp. SSHH-1E. While Bankura *et al*.^41^ used 10 mM of AgNO_3_ in AgNPs preparation using dextran as reducing agent.

Figure 4B represents the effect of X_1_ (initial medium pH level) and X_3_ (phycocyanin concentration), while X_2_ (AgNO_3_ concentration) and X_4_ (incubation period) were held at their zero levels (5 mM and 24 h; respectively). Maximum silver nanoparticles yield appeared at alkaline pH. The silver nanoparticles biosynthesis increases gradually by increasing phycocyanin concentration with increasing of initial medium pH. Then AgNPs gradually decreased at higher phycocyanin concentration.

AgNPs can obtained neither by grams of wet weight biomass of algal or cyanobacterial culture or cell-free culture liquid that suspended in mL(s) of 1 mM aqueous AgNO_3_, but in case of extracts like polysaccharides or phycobiliproteins, dry weight dissolved in mL (s) of deionized water then suspended in mL (s) of 1 mM aqueous AgNO_3_^16^.

Figure 4C represents the effect of X_1_ (initial medium pH level) and X_4_ (incubation period), while X_2_ (AgNO_3_ concentration) and X_3_ (phycocyanin concentration) were kept at their zero levels (5 mM and 1 mg/mL; respectively). It showed that the maximum silver nanoparticles yield appeared at alkaline medium pH. AgNPs biosynthesis increase with increasing incubation period and the initial medium pH, then decreasing of AgNPs occurred due to agglomeration of AgNPs at increased.

Biosynthesis of silver nanoparticles using *Ulva fasciata* ethyl acetate extract starts immediately after 2 min^39^, whereas AgNPs biosynthesis using cyanobacterial extracts have taken time ranged between 30 h to 360 h, *Microchaete* sp. NCCU-342 (30 h), *Spirulina* NCCU-477 (45 h), *Chroococcus* NCCU-207 (120 h), *Calothrix brevissema* NCCU-65(220 h), *Tolypothrix tenuis* NCCU-122 (300 h), *Oscillatoria* sp. NCCU-369 (360 h)^42^.

Figure 4D represents the effect of X_3_ (phycocyanin concentration) X_2_ and (AgNO_3_ concentration), while X_1_ (initial pH level) and X_4_ (incubation period) were kept at their zero levels (10 and 24 h; respectively). It showed that the maximum silver nanoparticles yield clearly situated close to the central point of both AgNO_3_ concentration and phycocyanin concentration.

Figure 4E describes the effects of X_4_ (incubation period) and X_2_ (AgNO_3_ concentration) on AgNPs biosynthesis using phycocyanin pigment, when X_1_ (initial medium pH level) and X_3_ (phycocyanin concentration) were kept at their zero levels (10 pH and 1 mg/mL; respectively). It showed that the maximum silver nanoparticles yield clearly situated close to the central point of both incubation period and AgNO_3_ concentration. Shameli *et al*.^43^ reported that the increasing in the time of reaction resulted in gradual increase in the intensity of the surface plasmon resonance peak until 24 h but after 48 h the surface plasmon resonance peak change to broad shape and intensity decreased, this phenomenon is related to the increased size and also agglomeration of silver nano-crystals.

Figure 4F the effect of X_3_ (phycocyanin concentration) and X_4_ (incubation period) on AgNPs biosynthesis when X_1_ (initial medium pH level) and X_2_ (AgNO_3_ concentration) were kept at their zero levels (10 pH and 5 mM; respectively). Analysis of Fig. 4F clearly suggests that the increasing of phycocyanin concentration with increasing incubation period, AgNPs biosynthesis increase till middle levels, however increase in phycocyanin concentration leads to decrease in AgNPs biosynthesis.

### Verification of the model

For determination accuracy of the model and verifying the optimal concentrations of the factors obtained from the optimization experiment, experiments were repeated in triplicates under the optimized conditions and compared with the predicted data. The experimental AgNPs was 1100.025 µg/mL, where the predicted value by the polynomial model was 1064.032 µg/mL. The verification revealed a high degree of accuracy of the model of more than 96.72%, indicating the model validation under the tested conditions. The optimized conditions of the variables for silver nanoparticles biosynthesis by using phycocyanin obtained from fitted model were initial pH level (10), AgNO_3_ concentration (5 mM), phycocyanin pigment concentration (1 mg/mL) and incubation period (24 h).

### Characterization of AgNPs synthesized using phycocyanin pigment extracted from *Nostoc linckia*

#### Energy-dispersive X-ray (EDX) spectroscopy

**Figure 5:**
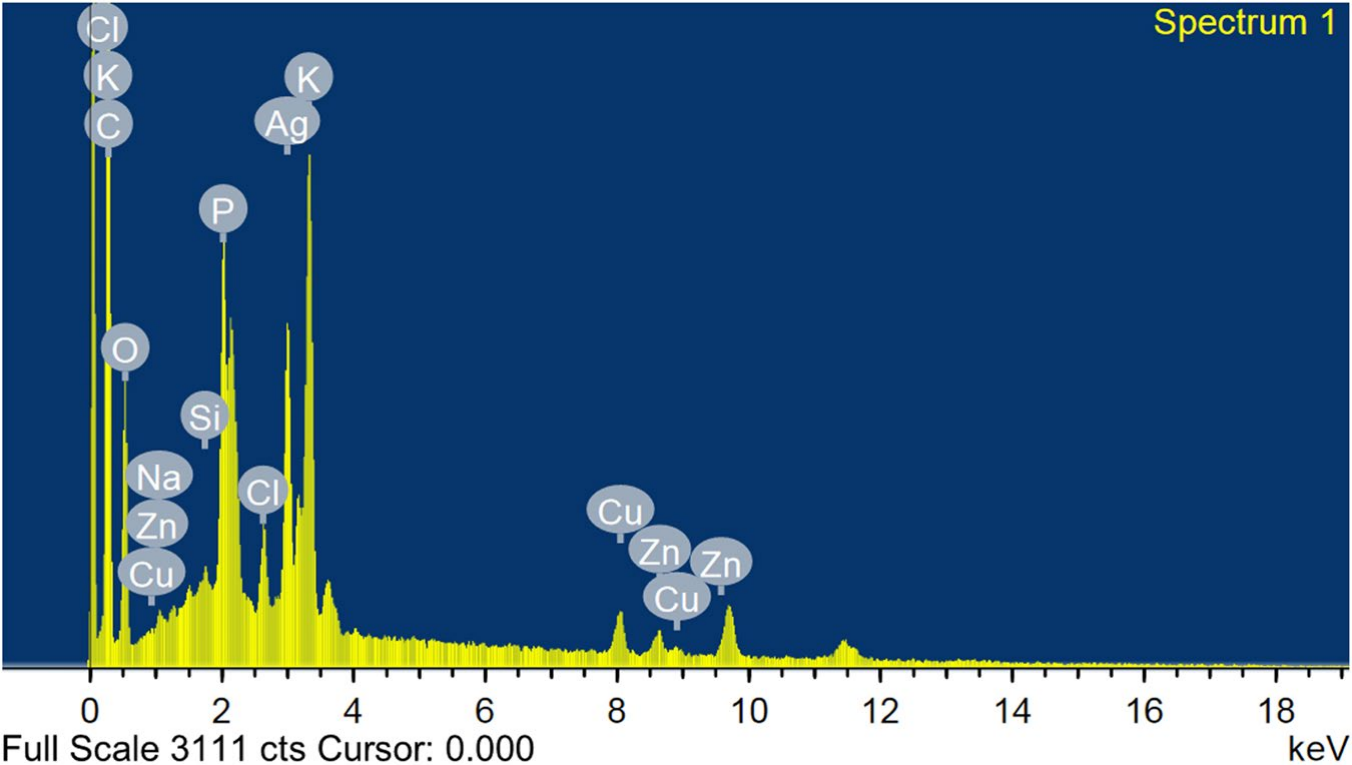
EDX spectrum recorded showing peak approximately near 3 keV confirming the presence of silver.

The sample powder of AgNPs was compressed to form tablets before analysis with EDX that assured that silver is the basic constituent element (Fig. 5). The crystal silver nanoparticles show typical optical observation peak approximately at 3 keV due to surface plasmon resonance and this result was compatible with Hebeish *et al*.^44^. Additionally, O and C elements peaks were observed. Peaks for Na, P, K, CL, Si, Cu and Zn were also observed.

#### X-Ray Diffraction (X-RD)

**Figure 6:**
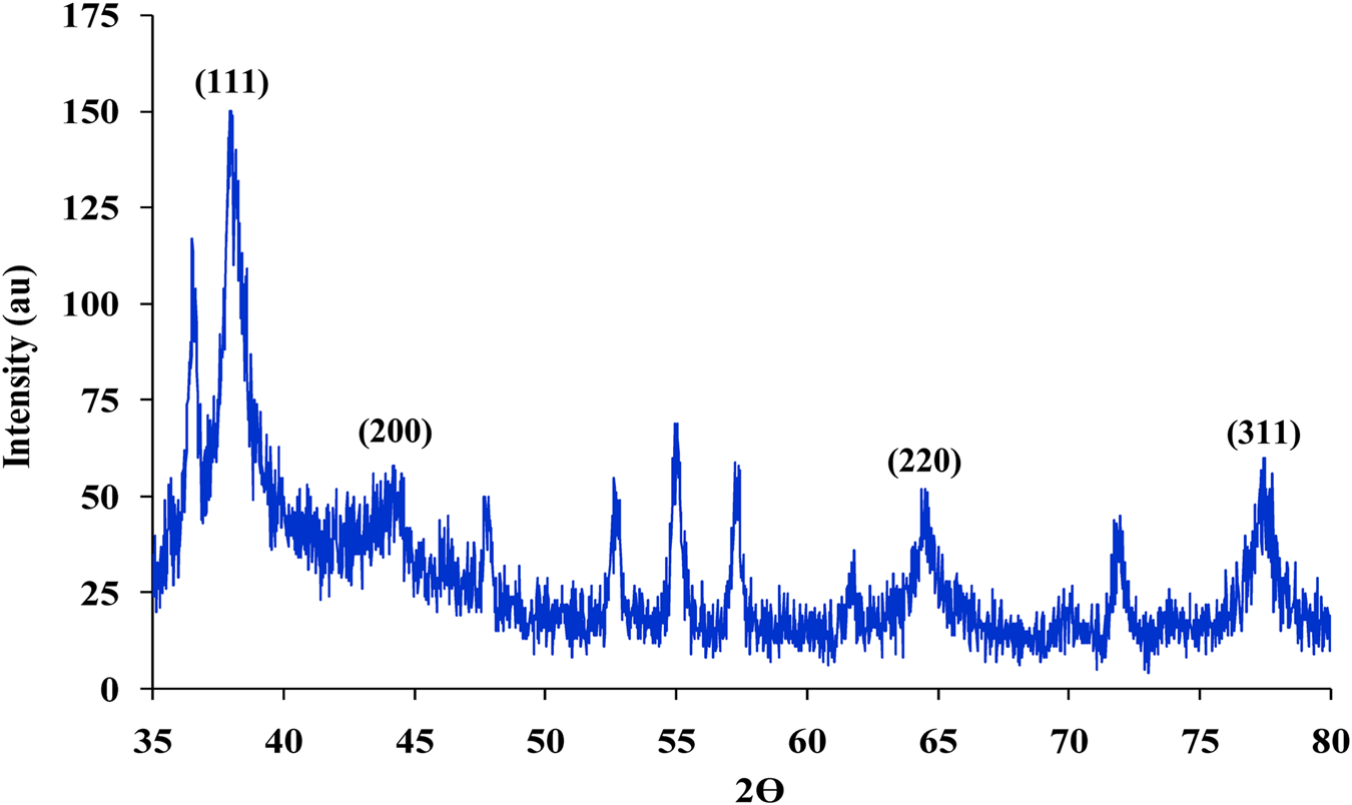
X-Ray Diffraction for silver nanoparticles synthesized by using phycocyanin pigment.

X-ray diffraction is now a common technique for the study of crystal structures and atomic spacing. The interaction of the incident rays with the sample produces constructive interference and a diffracted ray when conditions satisfy Bragg’s Law (*n*λ = 2*d* sin θ). The freeze–dried AgNPs was drop-coated using an X-ray powder diffractometer (Philips X’pert Pro, Panalytical) onto silica plate by applying many layer of small amount of samples on the plate with intermittent drying. The average size of AgNPs was estimated by the use of full width at half maximum (FWHM) of face-centered cubic (111) using the Debye–Scherrer equation, *K λ*/*β cos θ*, where *K* is the Scherrer constant with value from 0.9, *λ* is the wavelength of the X-ray, *β* is the full width at half maximum and *θ* is the Bragg angle in radians. XRD pattern for the silver nanoparticles is shown in Fig. 6, four well resolved diffraction peaks are observed. Diffraction features appearing at 2 theta (degree) as 37.9°, 44.3°, 64.5° and 77.3°, which correspond to the 111, 200, 220, and 311 planes of face centered cubic silver corresponding to the standard fcc structure; respectively, of silver is observed and compared with the standard powder diffraction card of Joint Committee on Powder Diffraction Standards (JCPDS), silver file No. 04–0783. Presence of these four intense peaks corresponding to the nanoparticles was in agreement with the Bragg’s reflections of silver identified with the diffraction pattern. The pattern of XRD suggests that the silver nanoparticles were essentially in the face centered cubic (fcc) structure and crystal in nature. The diffraction peaks observed for the silver nanoparticles indicates that these are crystalline in this size range. The XRD pattern of AgNPs (Fig. 6) comprised an amorphous peak and several crystallization peaks. AgNPs crystallite size determined from the highest intense refection (111) was found to be 3.24 nm.

#### Transmission electron microscopy

**Figure 7:**
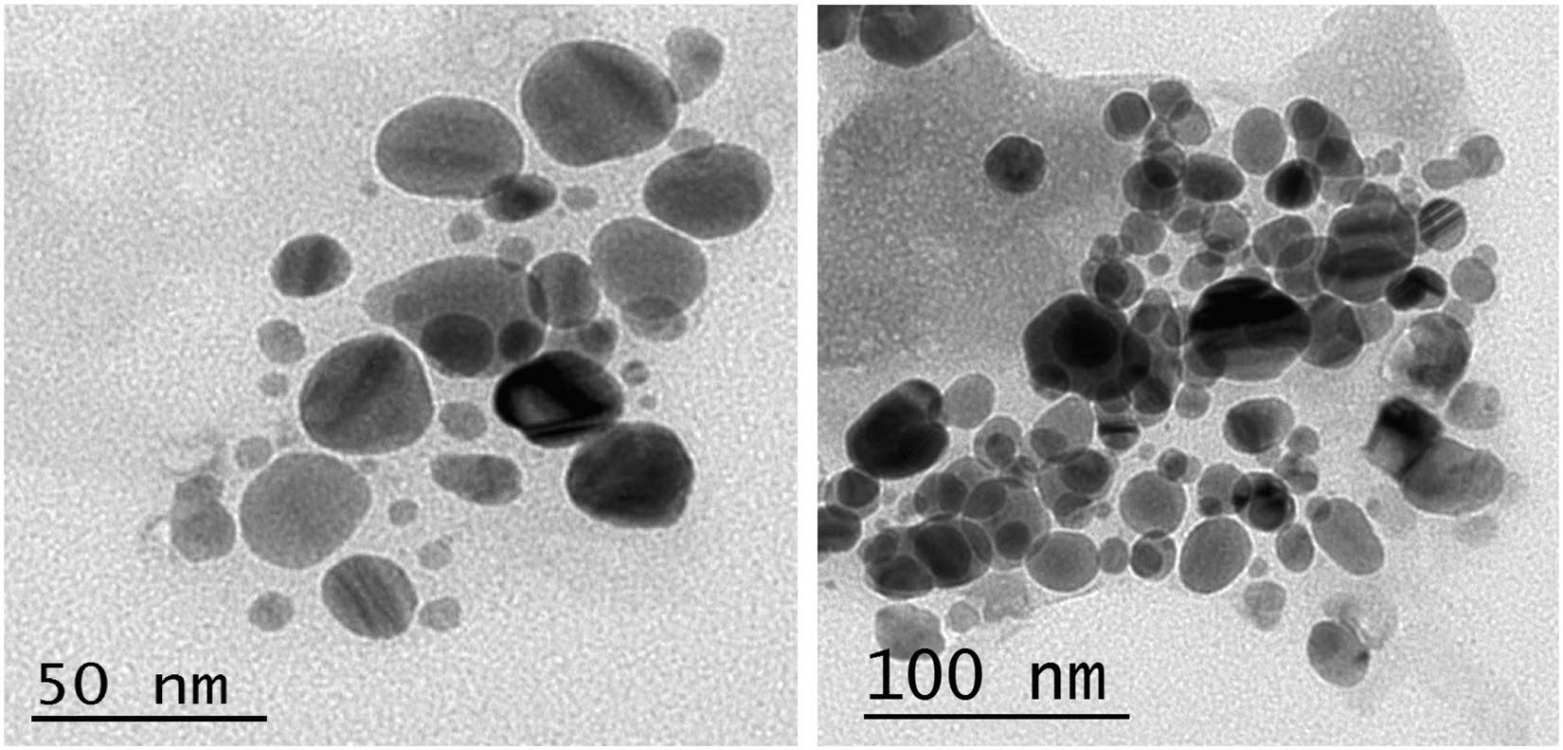
Transmition electron microscopy images of produced silver nanoparticles using phycocyanin pigment. Size-controlled silver nanoparticles synthesized over the range 9.39 to 25.89 nm.

Transmission electron microscopy (TEM) is a direct and effective approach to determine the particle size and morphology of the biosynthesized AgNPs, besides their dispersion uniformity. TEM was used for getting important information on primary nanoparticles size and morphology^45^. Transmission electron microscopy analysis of the biosynthesized AgNPs revealed the formation of spherical nanoparticles with a size range of 9.39 to 25.89 nm (Fig. 7). AgNPs is predominantly spherical in shape through microbial mediated synthesis^46^.

#### Fourier transformed infrared (FTIR) spectroscopy analysis

**Figure 8:**
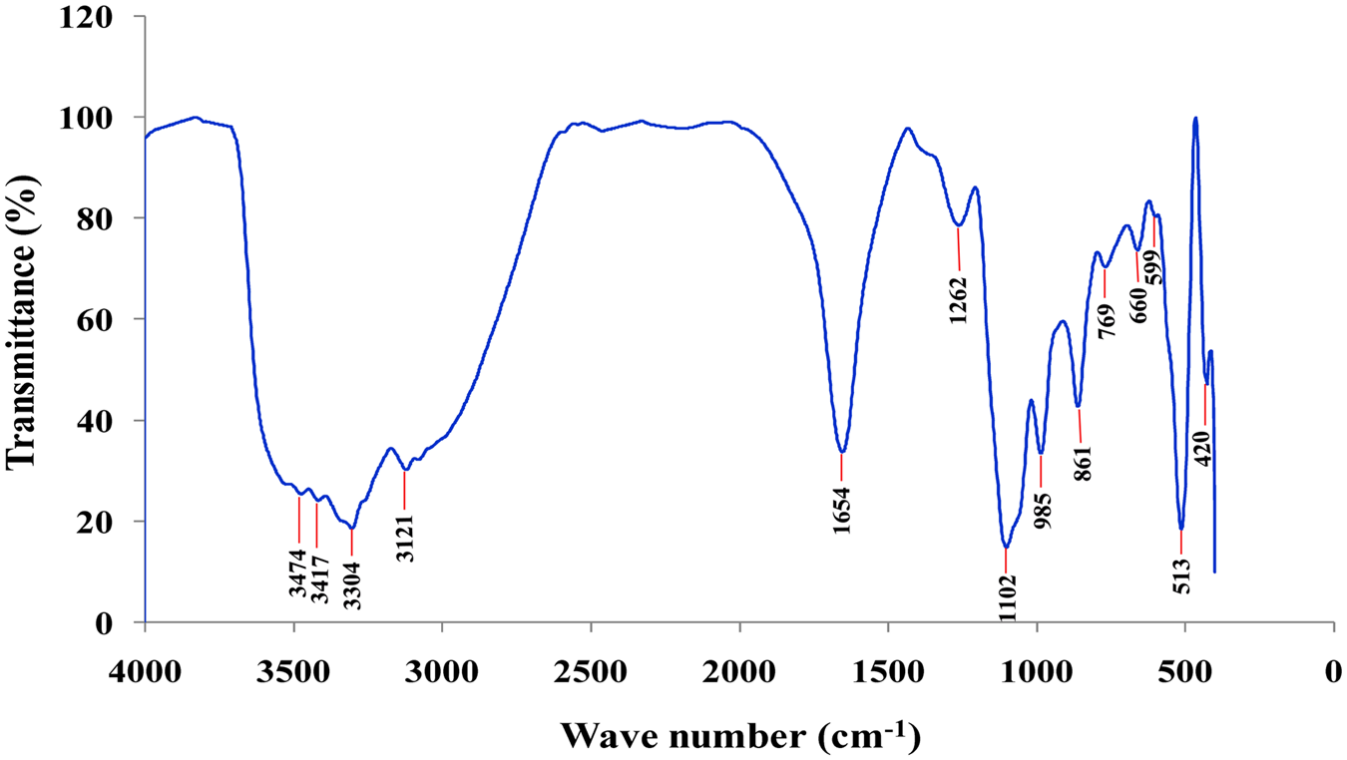
FTIR spectrum recorded by making KBr disc with synthesized silver nanoparticles by using phycocyanin pigment.

FTIR spectrum used for investigation of the functional groups and Ag nanoparticles formation mechanism particularly to identify possible interaction between silver precursor salt and protein molecules, leading the reduction of silver ions and stabilization of silver nanoparticles^47^. FTIR spectrum (Fig. 8) shows different 14 peaks positions at 3474, 3417, 3304, 3121, 1654, 1262, 1102, 985, 861, 769, 660, 599, 513 and 420 cm^−1^. The band at 3474 cm^−1^ could be assigned to N–H stretch found in aromatic amines, primary amines and amides^48^, and the peak at 3417 cm^−1^ attributed to O‒H stretching vibrations of phenol and alcohol^49^. The band at 3304 cm^−1^ attributed to ‒NH stretch present in polysaccharide and protein, ≡CH‒H stretch in acetylene and O‒H in oximes. The band at 3121 cm^−1^ assigned to NH_3_^+^ antisym stretch in amino acids present in extract^50^. The FTIR band at 1654 cm^−1^ assigned to C=O stretch with N‒H deformation found in primary amides II, C=O stretch in carbonyl groups^51^. The peak at 1262 cm^−1^ assigned to the presence of C–N stretching vibration of aromatic amines^52^. In addition, the presence of bands at 1102 cm^−1^ assigned to C=S stretching in thiocarbonyl compounds. It was shown a peak in the range of 985 cm^−1^ relating to C–N stretching vibration of primary amines that referring to possible involvement of primary amines during nanoparticles synthesis^53^ The peaks at 599 and 513 cm^−1^ assigned to C–C–CN nitrites, C–C=O bend ketones respectively. The FTIR band at 420 cm^−1^ assigned to CL–C=O in plane deformation of acid chlorides^50^. Thus, these biomolecules may be responsible for capping and efficient stabilization of synthesized nanoparticles. Theivasanthi *et al*.^47^ reported that proteins can bind to AgNPs through free amine groups of proteins which is compatible with our results.

FTIR spectrum supports the presence of a type of a protein on the surface of biosynthesized AgNPs, confirming that metabolically produced proteins acted as capping agents during production and prevented the reduced silver particles agglomeration. It has been suggested that stability of the AgNPs could be due to the presence of a proteinaceous capping agent that encapping and encapsulating them and forms a layer which protects the nanoparticles from agglomeration^54^.

#### The surface zeta potential distribution of silver nanoparticles

**Figure 9:**
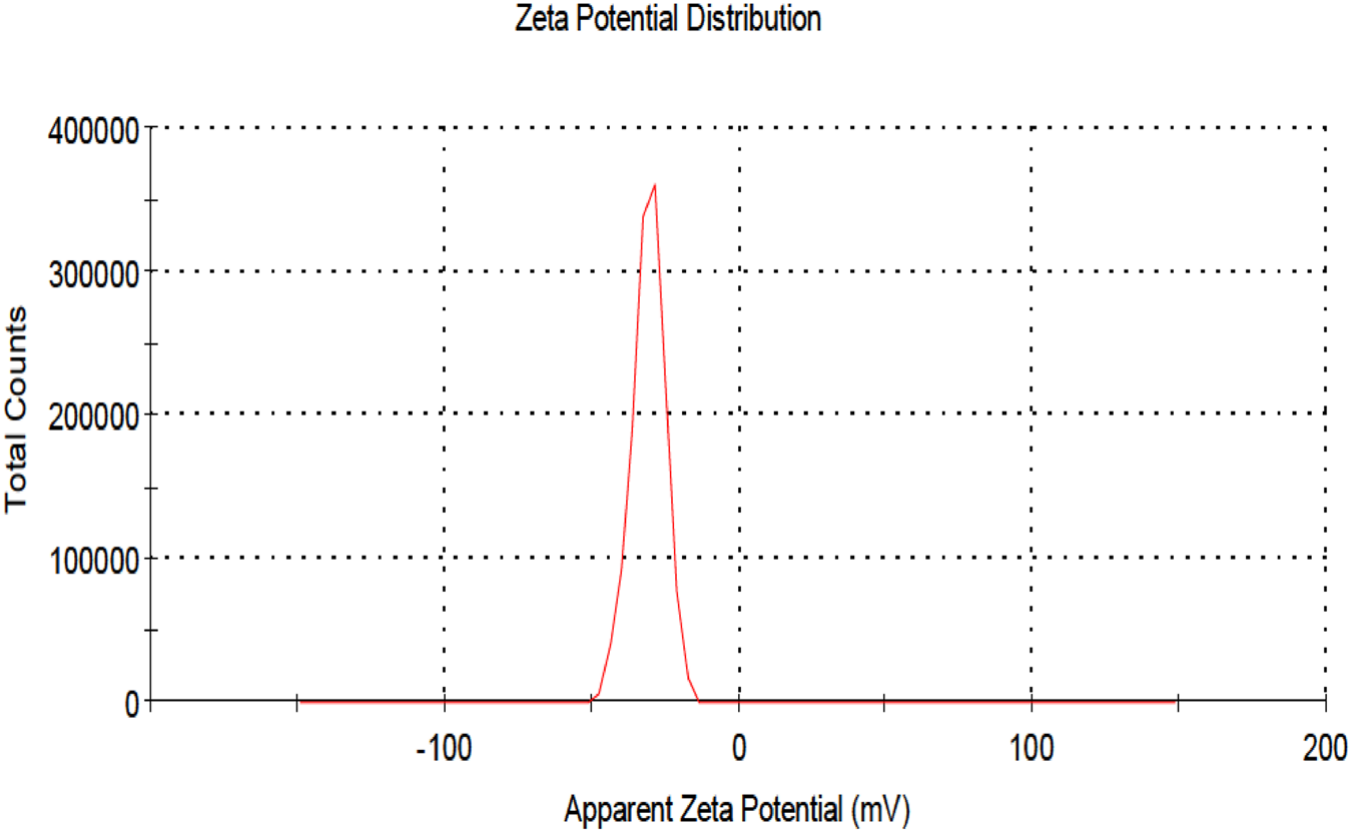
The zeta potential distribution graph showing negative zeta potential value for silver nanoparticles synthesized by using phycocyanin pigment.

In the research under study, zeta potential measurement (Fig. 9) is −31.8 mV at standard deviation 5.37 mV with conductivity 0.420 mS/cm. The biosynthesized AgNPs are consider strongly anionic which is important in biological application according to Aiad *et al*.^55^. Typically, nanoparticles with zeta potentials greater than 20 mV or less than −20 mV have sufficient electrostatic repulsion to remain stable in solution. Salehi *et al*.^56^ suggested that the greater negative surface charge potential (−31 mV) value may be attributed to the effective functional constituents as capping agents present in the ethanol extract of *Artemisia marschalliana* Sprengel aerial part extract.

### Applications of the synthesized AgNPs

#### Antimicrobial activity of synthesized AgNPs

**Figure 10:**
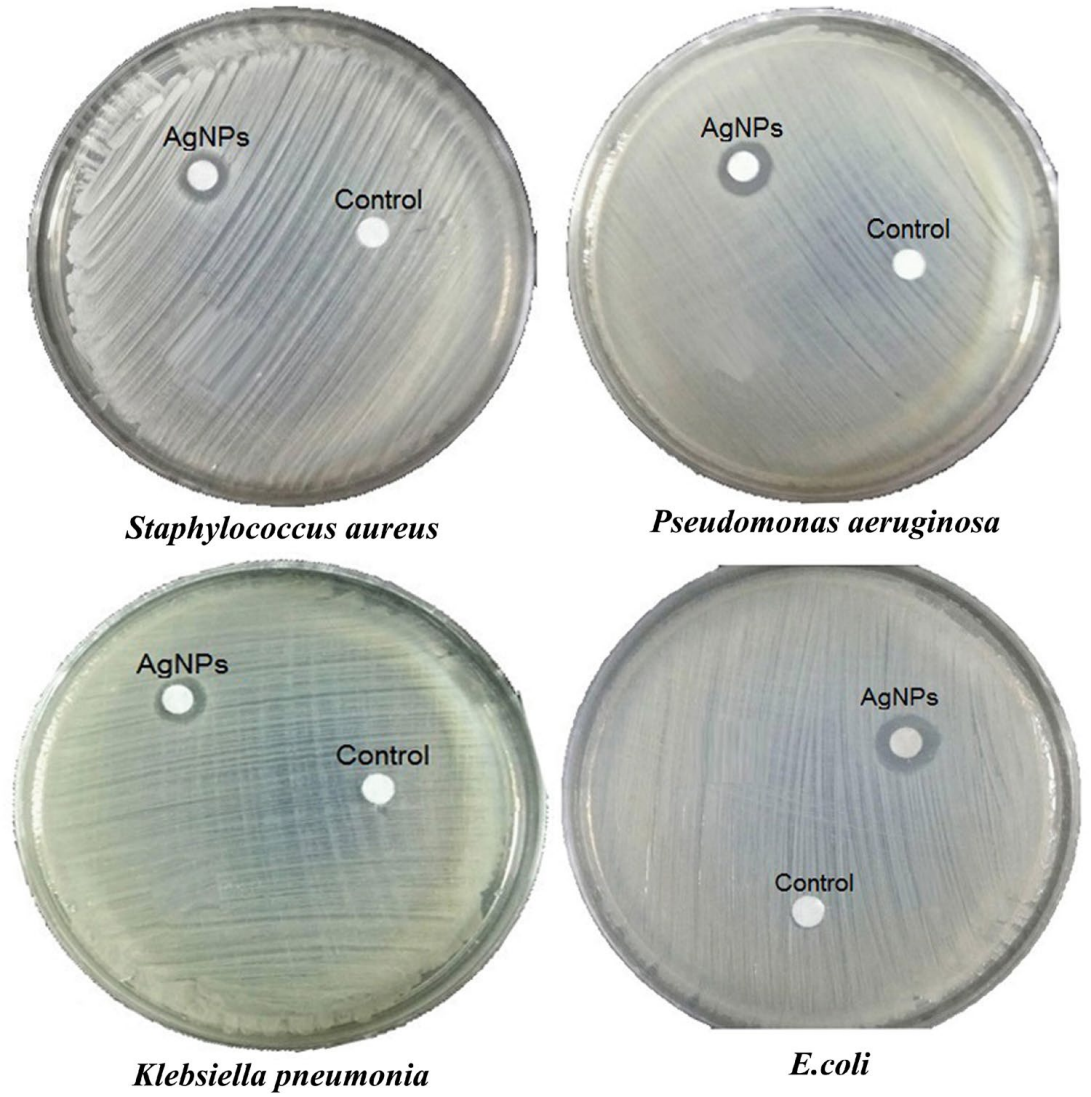
Antibacterial activity of silver nanoparticles produced by using phycocyanin pigment against bacterial species.Inhibition zones were 9, 10, 10, 9 mm against *Staphylococcus aureus*, *Pseudomonas aeruginosa*, *E. coli* and *Klebsiella pneumonia*; respectively.

The antimicrobial activity of silver nanoparticles produced by using phycocyanin pigment was evaluated against bacterial species: Gram-positive bacteria (*Staphylococcus aureus*), Gram-negative bacteria (*Pseudomonas aeruginosa*, *Klebsiella pneumonia* and *E. coli*) by the disc-diffusion method (Fig. 10). A control (5 mM AgNO_3_) was also maintained in each plate. The diameter of inhibition zones around each disc is measured. The highest antibacterial activity was observed against *E. coli* which was similar to the antibacterial activity to *Pseudomonas aeruginosa* (10 mm) whereas a low activity was found against *Klebsiella pneumonia* (9 mm), which also similar to the antibacterial activity against *Staphylococcus aureus*. This result is compatible with previous studies that examined the antimicrobial activity of AgNPs against *Staphylococcus aureus* ^57^. Biosynthesized silver nanoparticles showed significant antibacterial effect on both Gram classes of bacteria. There are many ways for AgNPs to interact with microorganisms and damage them. Silver ions released from silver nanoparticles when come in contact with bacterial cells may deactivate the production of some enzymes and cellular proteins necessary for adenosine tri-phosphate (ATP) synthesis or influence on the bacterial DNA replication functions^58^. In other researches, silver ions may disrupt the working of membrane-bound enzymes of the respiratory chain^59^. The antibacterial activity of silver nanoparticles are affected by the particles size and the smaller the particles, the greater the antibacterial efficacy^60^. Other studies showed that the bacterial damages by rupture of plasma membrane or by blocking respiration in association with oxygen and sulfhydryl (S H) groups on the cell wall to form R S S R bonds thereby, leading to exhaust of intercellular ATP and cell death^61^. It is reported that the electrostatic interaction between the positive charge on the silver ion and the negatively charged cell membrane of microorganisms is the reason for antimicrobial activity^62^.

#### Anti-hemolytic assay

Erythrocyte hemolysis is the disruption of the integrity of its membrane that causes releasing of haemoglobin into the surrounding blood plasma. Erythrocytes are oversensitive to oxidative disruption due to membrane high poly-unsaturated fatty acid content and high concentration of oxygen and hemoglobin which are important promoter of oxidative processes^63^. The free radical can produce lipid peroxyl radicals which attack the lipids membrane and converted into lipid hidroperoxides leading to adverse effects on membrane structure and function^64^. AAPH is consider a soluble hydrophilic azo-compound at which generates 2-amidinopropyl radicals called C-radicals through a process depending on specific temperature. These C-radicals reacts with oxygen molecules (if present) and generate peroxyl radicals and hemolysis increases by time with increasing the incubation period of erythrocyte with AAPH^65^.

The addition of AgNPs to erythrocyte exposed to the free radicals of AAPH exhibited low erythrocyte hemolysis of 7.8% (i.e AgNPs exhibit anti-hemolytic activity with 92.2% inhibition) which was very low as compared with complete (100%) erythrocyte hemolysis which occurs at the incubation of erythrocyte and AAPH without AgNPs. AgNPs can prevent the oxidative damage in healthy erythrocytes. Standard ascorbic acid has showed percentage erythrocyte hemolysis of 4.0%. The proposal anti hemolytic activity of AgNPs is due to the presence of O‒H group present in phenolic compounds or Ar‒OH or ‒NH_2_ in aromatic amines compounds surrounded the Ag ion possess an important antioxidant activity which prevent oxidation by the free radicals^66^. Mallesha *et al*.^67^ proposed that the anti-hemolytic activity of AgNPs is that AgNPs include -NH_2_ in aromatic amines, primary amines and amides which is the functional group of amine compound that exhibit anti-hemolytic activity with 58% inhibition compared to standard quericitin with 74% inhibition in the synthetic compound (4-nitro-benzylidene)-pyridin-3-ylmethyl amine.

#### *In vitro* anti-cancer activity

**Figure 11:**
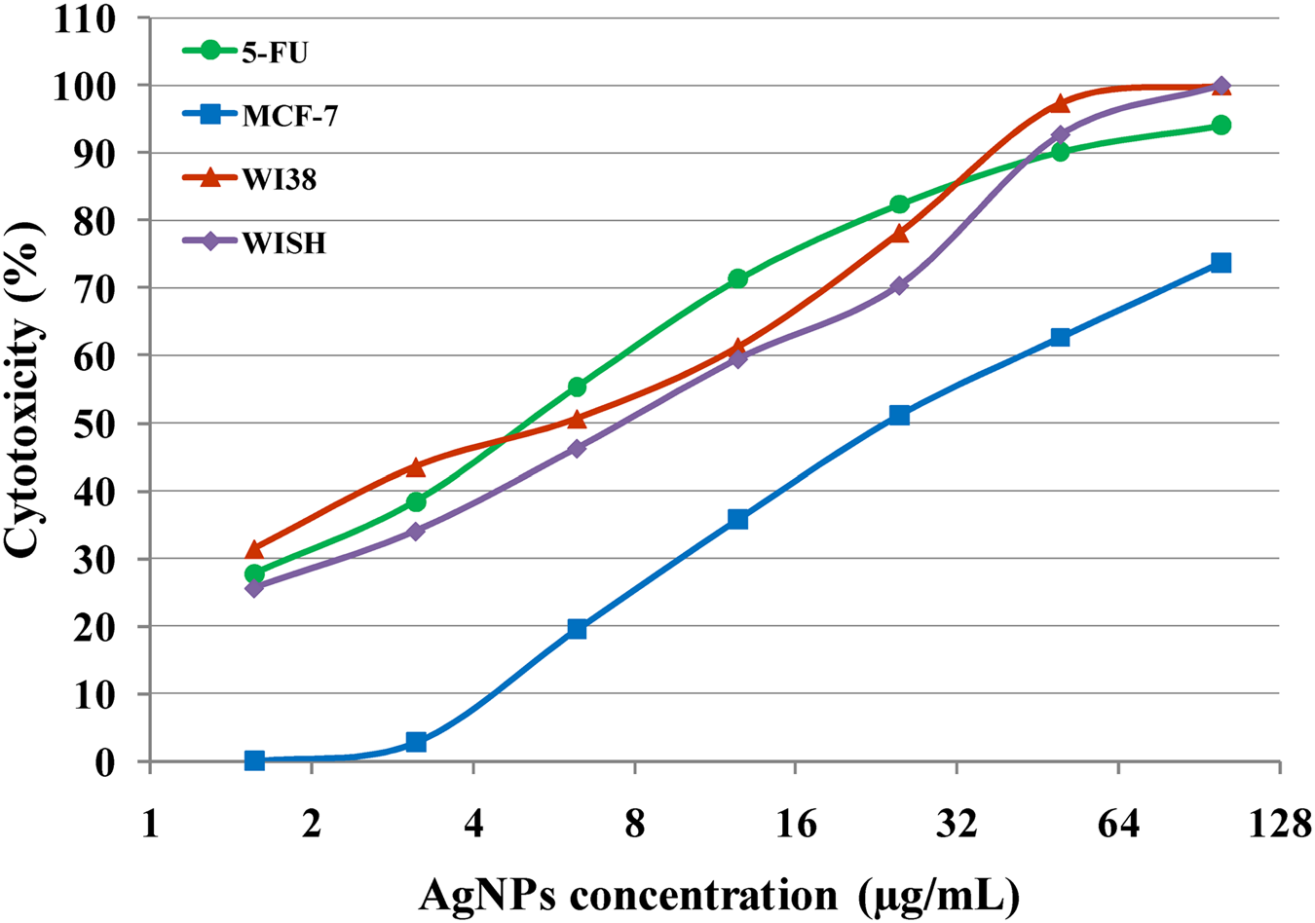
*In vitro* anti-cancer activity of various concentrations of AgNPs on mammary gland breast cancer cell line (MCF-7), human lung fibroblast (WI38) and human amnion (WISH) the cell lines . 5-fluorouracil was used as a standard anticancer drug for comparison.

Silver nanoparticles have earned a great reputation in the field of nanomedicine due to their unique properties which obvious therapeutic potential in the diagnosis and treatment of some human cancer types^68^. In the research under study, the cytotoxic effect of silver nanoparticles against mammary gland breast cancer (MCF-7) cell line and cytotoxicity (%) was carried out *in vitro* by MTT assay and compared with the standard 5- 5-5-fluorouracil that commercially available as anticancer drug. Comparison of the cytotoxicity of synthesized AgNPs (1.56, 3.12, 6.25, 12.5, 25, 50, 100 µg/mL) and similar concentration in 5-FU disclosed similar mortality rate. Cytotoxicity rate against MCF-7 cell lines increases with increase in concentration of silver nanoparticles (Fig. 11). The inhibitory effect was observed after 48 h of incubation. The results were exhibited as growth inhibitory concentration (IC_50_) values, which represent the compounds concentrations required to produce a 50% inhibition of cell growth after 48 h of incubation, compared to untreated controls.

The IC_50_ of cell inhibition of silver nanoparticles was observed at 27.79 ± 2.3 µg/mL of AgNPs against the breast cancer MCF-7 cell lines. The IC_50_ of the cell inhibition of silver nanoparticles against human lung fibroblast (WI38) and human amnion (WISH) cell lines were observed at 31.78 ± 2.2 and 32.97 ± 1.7 µg/mL respectively. So the produced AgNPs exhibit stronger cytotoxic effect on the breast cancer MCF-7 than WI38 and WISH normal cell lines.

### Proposed mode of action of AgNPs on MCF-7 cells

**Figure 12:**
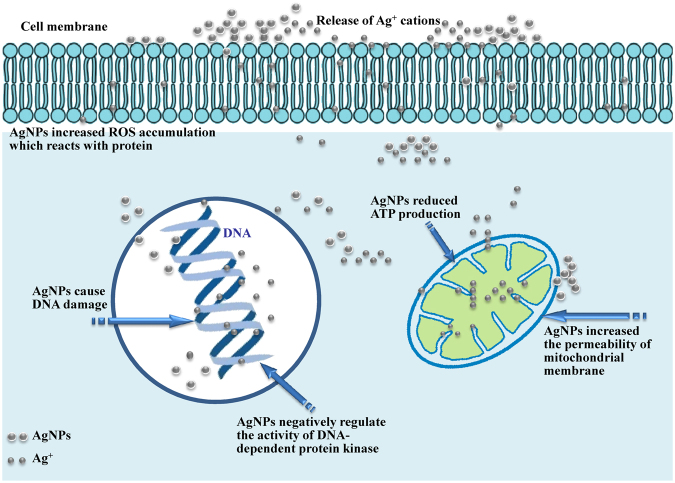
Schematic representation of the proposed mode of action of AgNPs on MCF-7 cells.

Schematic representation of the proposed mode of action of AgNPs on MCF-7 cells was demonstrated in Fig. 12. AgNPs uptaken by tumor cells (MCF-7) are catabolized forming amino acids and Ag ions^69^. The cytotoxic properties of the nanoparticles due to the release of Ag+ cations which interact with cells and intracellular macromolecules like proteins and DNA. AgNPs have shown to cause DNA damage^70^ and increased mitochondrial membrane permeability^71^.Cellular uptake of nanoparticles which subsequently captured free electrons and increased the synthesis and accumulation of intercellular reactive oxygen species (ROS) which reacts with protein and cause oxidative stress^72^. This process leads to the partial or permanent loss of structure and/or function of cellular protein. It further reduced adenosine triphosphate (ATP) production^73^. In addition to enhancing the generation of potentially damaging radicals, AgNPs have also been shown to negatively regulate the activity of DNA-dependent protein kinase, a key enzyme involved in DNA damage repair via nonhomologous end joining^74^. Cell damage by silver nanoparticles may be due to loss of cell membrane integrity, apoptosis and oxidative stress.

The differential response of breast cancer cells to AgNPs induced hyperthermia, which implies AgNPs to be effective photothermal agents^75^. Apoptosis could be activated through mitochondrial dysfunction, which potentially inhibits the proliferation of MCF-7 cells^76^. Ranjitham *et al*.^77^ studied *in vitro* cytotoxicity of the AgNPs against MCF-7 breast cancer cell line at different concentrations. The samples showed a considerable cytotoxicity against the MCF-7cell line. It was reported that the toxicity of AgNPs increase with increase in concentration.

#### *In vivo* cytotxicity of AgNPs on Ehrlich ascites tumor

**Figure 13:**
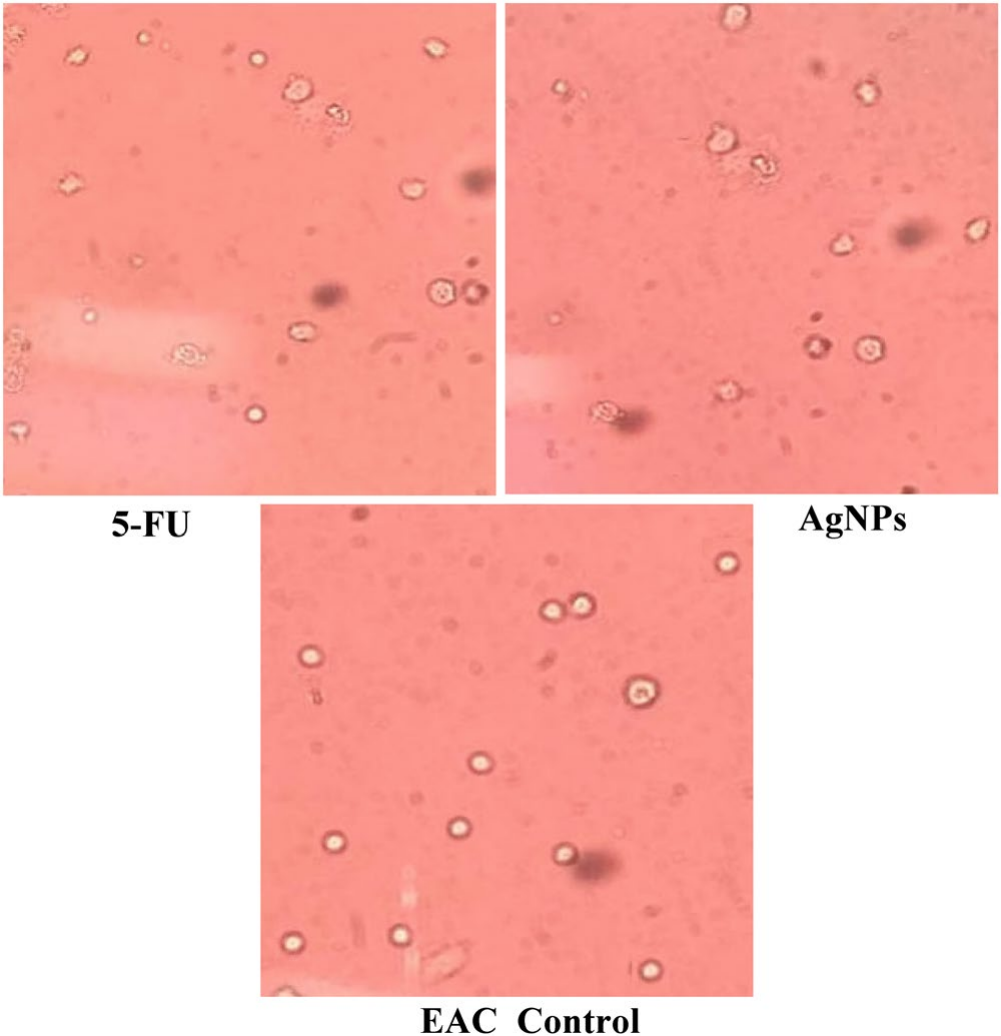
Microscopy images demonstrate the cytotoxic effect of AgNPs and 5-FU on EAC cells, cell damage by AgNPs due to loss of cell membrane integrity and apoptosis.

The present investigation was carried out to evaluate the antitumor activity of AgNPs in Ehrlich ascites carcinoma bearing mice (Table 4 and Fig. 13). RBC count and hemoglobin content in the Ehrlich ascites carcinoma (EAC) control group (3.36 ± 0.36 10^6^/mm^3^ and 8.10 ± 0.79 g/dl; respectively) was decreased as compared to normal control group (5.23 ± 0.28 10^6^/mm^3^ and 13.53 ± 0.65 g/dl; respectively). Treatment with AgNPs at the dose of 5 mg/kg body weight increased the hemoglobin content and RBC count (13.86 ± 1.10 g/dl and 4.71 ± 0.42 10^6^/mm^3^; respectively which is higher than treatment with 5-FU that showed hemoglobin content and RBC count (12.97 ± 1.03 g/dl and 4.52 ± 0.51 10^6^/mm^3^; respectively (Table 4). Also the total WBC counts was found to be increased in EAC control group when compared with normal group, however total WBC counts after treatment with AgNPs (8.45 ± 1.16 10^3^/mm^3^) was found to be decreased than in the EAC bearing mice (15.06 ± 1.34 10^3^/mm^3^) and mice treated with 5-FU (9.32 ± 1.01 10^3^/mm^3^) (Table 4). Decreases in tumor cell count (12.41 ± 8.43 10^6^/mL), body weight (26.5 ± 2.8 g) and tumor volume (1.3 ± 1.2 mL) were observed in AgNPs treated animals when compared to EAC- bearing mice which showed tumor cell count (49.15 ± 9.6210^6^/mL), body weight (37.7 ± 3.1 g) and tumor volume (7.6 ± 1.8 mL) (Table 4).

**Table 4:** Effect of AgNPs and 5-FU on hematological parameters, tumor parameters (volume and cell count) and body weight of EAC bearing mice.

Group	Hb (g/dl)	RBC Count 10^6^/mm^3^	Total WBC 10^3^/mm^3^	Tumor volume (mL)	Tumor cell count (106/mL)	Body Wt. (g)
Normal Control	13.53 ± 0.65	5.23 ± 0.28	4.97 ± 0.93	—	—	24.2 ± 1.9
EAC Control	8.10 ± 0.79	3.36 ± 0.36	15.06 ± 1.34	7.6 ± 1.8	49.15 ± 9.62	37.7 ± 3.1
5-FU	12.97 ± 1.03	4.52 ± 0.51	9.32 ± 1.01	1.5 ± 0.6	13.24 ± 7.10	28.6 ± 2.3
AgNPs	13.86 ± 1.10	4.71 ± 0.42	8.45 ± 1.16	1.3 ± 1.2	12.41 ± 8.43	26.5 ± 2.8

The results clearly indicate that AgNPs has a capacity to inhibit the growth of tumor induced by EAC cell line in the experimental animals. The morphology of AgNPs formed by phycocyanin pigment extracted from *Nostoc linckia* showed that the size range is 9.39 to 25.89 nm. These small sizes obtained silver particles have capability of distribution and accumulation in tumor cells after its injection at the site of tumor. Devi *et al*.^78^ reported that the cytotoxic effect is inversely proportional to the size of AgNPs.

Once a tumor grows to a certain size, it needs nutrients and oxygen from the blood to grow and spread. The tumor sends chemical signals that stimulate the growth of new blood vessels that carry the blood to it, growth and metastasis of tumor depend on angiogenesis^79^. Cancer treatments with AgNPs block angiogenesis, limited angiogenesis occurs after AgNPs treatment. The tumor starved and dies if it cannot get enough nutrients and oxygen. Martins *et al*.^80^ reported that compounds having anti-angiogenic properties are known for their ability to block abnormally expressing signaling proteins. Antony *et al*.^79^ reported that anti-angiogenesis occurs after AgNPs treatment of DAL tumor cells. Also, AgNPs have a cytotoxic effect on EAC bearing mice by production of lipid peroxidation and free radicals in tumor tissues that proved by El Bialy *et al*.^81^.

Treatment with AgNPs at the dose of 5 mg/kg body weight significantly inhibited the tumor cells volume, tumor cells count and tumor weight. In case of control group, a regular rapid increase in ascetic tumor volume was observed. Ascitic fluid is the direct nutritional source for tumor cells and a rapid increase in ascetic fluid with tumor growth would be a means to meet the nutritional requirement. It may be concluded that AgNPs decreases the nutritional fluid volume and arresting the tumor growth. Thus AgNPs possess antitumor activity against EAC bearing mice. Body weight of Ehrlich ascites carcinoma bearing mice inoculated with AgNPs was observed to be decreased compared to Ehrlich ascites carcinoma bearing mice which could be positively correlated with the ascites fluid volume^79^. Anemia is the main problems in cancer treatment which encountered in tumor bearing mice is mainly due to reduction in RBC or haemoglobin percentage, and this may occur either due to iron deficiency or due to hemolytic or myelopathic conditions^82^.

## Materials and Methods

### Organism and culture conditions

The investigating cyanobacterium, *Nostoc linckia*, was previously collected from garden soil sample in Dakahlia, Egypt. One inoculum of alga was cultured in BG-11 medium^83^. The fresh culture of the alga was prepared in 500 mL Erlenmeyer flasks containing 250 mL of BG-11 medium and inoculated with 10 mL of 14 days old culture. The flasks were incubated at 25 ± 2 °C for 28 days under illumination (600–800 lux) by 36 W white fluorescent lamp to achieve a maximum grow rate. Collecting all the fresh biomass from the medium for being freeze drying by Freeze dryer (SIM international, USA, FD8-8T).

### Extraction of phycocyanin

Phycocyanin is pigment-protein complexes from the light-harvesting pycobiliprotein. It was extracted by adding 100 mg of freeze-drying biomass to a solution of 100 mL phosphate buffer (pH7) and successive freezing and thawing process was applied for about one week to complete extraction of the pigment. The extract was filtered to remove cell depresis.

### Extracellular synthesis, precipitation and centrifugation of AgNPs

1 mL of AgNO_3_ solution (5 mM) was added to 19 mL of phycocyanin pigment extracted from *Nostoc linckia*. pH was adjusted to pH 10 using NaOH (1 M) and incubated within closed system under direct illumination (2400–2600 Lux) for 24 h at room temperature. The biosynthesis of AgNPs was indicated by color change to dark brown. After incubation period, the bio reduction reaction was monitored by visual color change and UV– visible absorbance of the reaction mixture in the range of 200–800 nm on UV-Vis spectrophotometer (ATI Unicam 5625 UV/VIS Vision Software V3.20). AgNO_3_ solution was maintained as a control. AgNPs were obtained by centrifugation (MIKRO 120 Hettich Zentrifugen D-78532 Tuttlingen Germany) at 10,000 *g* for 10 min. The supernatant was discarded and then the precipitated AgNPs were washed with sterile deionised water for 10 times for complete purification. After washing, the precipitated AgNPs were freeze-dried to obtain the dry AgNPs used for both characterization and applications.

### Face-centered central composite design (FCCD)

In this study, the effect of four process variables namely initial pH level (X_1_), AgNO_3_ concentration (X_2_), phycocyanin pigment concentration (X_3_) and incubation period (X_4_) on biosynthesis of silver nanoparticles was studied and optimized using face-centered central composite design (FCCD). FCCD is an effective design that is used for sequential experimentation and provides reasonable amount of information for testing the goodness of fit and does not require large number of design points thereby reducing the overall cost associated with the experiment^84^. In this study, the experimental plan consisted of 30 trials and the independent variables were studied at three different levels, low (−1), middle (0) and high (+1). The center point was repeated six times in order to evaluate the curvature and the experiment replication facilitated the pure error estimation, so that the significant lack of fit of the models could be predicted. All the experiments were done in duplicate and the average of AgNPs obtained was taken as the dependent variable or response (Y). The experimental results of FCCD were fitted via the response surface regression procedure using the following second order polynomial equation:

2 $$Y={\beta }_{0}+\sum _{i}{\beta }_{i}{X}_{i}\,+\,\sum _{ii}{\beta }_{ii}{X}_{i}^{2}+\sum _{ij}{\beta }_{ij}{X}_{i}{X}_{j}$$

In which Y is the predicted response, β_0_ is the regression coefficients, β_i_ is the linear coefficient, β_ii_ is the quadratic coefficients, β_ij_ is the interaction coefficients), and X_i_ is the coded levels of independent variables.

However, in this study, the independent variables were coded as X_1_, X_2_, X_3_ and X_4_. Thus, the second order polynomial equation can be presented as follows:

3 $$\begin{array}{rcl}{\boldsymbol{Y}} & = & {{\boldsymbol{\beta }}}_{{\bf{0}}}+{{\boldsymbol{\beta }}}_{{\bf{1}}}{{\boldsymbol{x}}}_{{\bf{1}}}+{{\boldsymbol{\beta }}}_{{\bf{2}}}{{\boldsymbol{x}}}_{{\bf{2}}}+{{\boldsymbol{\beta }}}_{{\bf{3}}}{{\boldsymbol{x}}}_{{\bf{3}}}+{{\boldsymbol{\beta }}}_{{\bf{4}}}{{\boldsymbol{x}}}_{{\bf{4}}}+{{\boldsymbol{\beta }}}_{{\bf{12}}}{{\boldsymbol{x}}}_{{\bf{1}}}{{\boldsymbol{x}}}_{{\bf{2}}}\\  &  & +{{\boldsymbol{\beta }}}_{{\bf{13}}}{{\boldsymbol{x}}}_{{\bf{1}}}{{\boldsymbol{x}}}_{{\bf{3}}}+{{\boldsymbol{\beta }}}_{{\bf{14}}}{{\boldsymbol{x}}}_{{\bf{1}}}{{\boldsymbol{x}}}_{{\bf{4}}}+{{\boldsymbol{\beta }}}_{{\bf{23}}}{{\boldsymbol{x}}}_{{\bf{2}}}{{\boldsymbol{x}}}_{{\bf{3}}}+{{\boldsymbol{\beta }}}_{{\bf{24}}}{{\boldsymbol{x}}}_{{\bf{2}}}{{\boldsymbol{x}}}_{{\bf{4}}}\\  &  & +{{\boldsymbol{\beta }}}_{{\bf{11}}}{{\boldsymbol{x}}}_{{\bf{1}}}^{{\bf{2}}}+{{\boldsymbol{\beta }}}_{{\bf{22}}}{{\boldsymbol{x}}}_{{\bf{2}}}^{{\bf{2}}}+{{\boldsymbol{\beta }}}_{{\bf{33}}}{{\boldsymbol{x}}}_{{\bf{3}}}^{{\bf{2}}}+{{\boldsymbol{\beta }}}_{{\bf{44}}}{{\boldsymbol{x}}}_{{\bf{4}}}^{{\bf{2}}}\end{array}$$

### Statistical analysis

Design Expert® 7.0 software version 7 (Stat-Ease Inc., USA) for Windows was used for the experimental designs and statistical analysis. The statistical software package, STATISTICA software (Version 8.0, StatSoft Inc., Tulsa, USA) was used to plot the three-dimensional surface plots, in order to illustrate the relationship between the responses and the experimental levels of each of the variables utilized in this study.

### Characterization of silver nanoparticles

#### UV–visible spectral analysis

The biosynthesized AgNPs were monitored by measuring the UV-Vis spectrum after 24 hours of reaction. A small aliquot was drawn from the reaction mixture and a spectrum was taken on a wavelength from 200 to 800 nm on UV-Vis spectrophotometer (ATI Unicam 5625 UV/VIS Vision Software V3.20) at Spectrum Unit of Faculty of Science, Mansoura University, Egypt. AgNO_3_ solution was used as a control throughout the experiment.

#### Energy-dispersive X-ray (EDX) spectroscopy analysis

Energy dispersive X-ray analysis (EDX) was carried out with the scanning electron microscope (Oxford X-Max 20) with secondary electron detectors at an operating voltage of 20 kV at Electron Microscope Unit, Mansoura University, Mansoura, Egypt.

#### X-ray diffraction analysis (XRD)

X-ray diffraction analysis was carried out by using an X-ray diffractometer (Philips X’pert Pro, Panalytical) having CuKa (k = 1.54 A°) radiation and a programmable divergence slit. The voltage and current of the X-ray source were 40 kV and 20 mA; respectively. The sample was drop-coated onto silica plate by applying many layer of small amount of biosynthesized AgNPs on the plate with intermittent drying. This leads to a thick coat of AgNPs to be examined with monitoring the diffraction angle from 5° to 80° (2θ).

#### Transmission electron microscopy (TEM) analysis

TEM produce a high-resolution, two-dimensional image which utilizes energetic electrons to provide morphologic, compositional and crystallographic information on the biosynthesized AgNPs. The morphology and size of the AgNPs synthesized using phycocyanin extracted from *Nostoc linckia* was visualized using ultra high resolution transmission electron microscope (JEOL-JEM-100 CXII instrument) operating at an accelerating voltage of 200 kV. Samples were prepared by drying a drop of the washed AgNPs dispersion onto the carbon-coated copper grid and dried under infrared lamp prior to examination at Electron-Microscope-Unit of Mansoura University, Egypt.

#### Fourier-Transform Infra-Red (FTIR) spectroscopy analysis

The synthesized AgNPs sample was freeze dried and diluted with potassium bromide (in the ratio of 1:100) to make a pellet. The FTIR spectrum of sample was recorded on a FTIR instrument (Thermo Scientific Nicolet iS10 FT-IR spectrometer) At Spectrum Unit of Faculty of Science, Mansoura University, Mansoura, Egypt. The measurement was carried out in the range of 500*–*4000 cm^−1^ at a resolution of 4 cm^−1^.

#### The surface zeta potential distribution of silver nanoparticles

Laser Doppler Micro-electrophoresis is the technique used to measure zeta potential and zeta size. The particle size distribution and surface charge of AgNPs were determined using particle size analyzer (Zeta sizer nano ZS90, Malvern Instruments Ltd., U.K.) at 25 °C with 90° detection angle at Electron Microscope Unit of Mansoura University, Mansoura, Egypt.

### Applications of the synthesized AgNPs

#### Antimicrobial activity of AgNPs by disk-diffusion method

Antimicrobial activity of AgNPs biosynthesized by phycocyanin was tested against bacterial pathogens of Gram-positive (*Staphylococcus aureus*), Gram-negative bacteria (*Pseudomonas aeruginosa, E. coli* and *Klebsiella pneumonia*) using disk- diffusion method on nutrient agar plates. Sterile filter paper discs (6 mm in diameter) were saturated with 20 µl (1000 µg/mL) AgNPs solution, disc saturated with 5 mM AgNO_3_ solution was used as control. The plates were incubated at 37 °C for 24 h and then were examined for the presence of inhibition zones. The diameters of inhibition zones around the discs were measured and the mean value for each organism was recorded and expressed in millimeter.

#### Anti-hemolytic activity of AgNPs

The anti-hemolytic activity of AgNPs was determined using blood obtained from rats by cardiac puncture and collected in heparinized tubes. Erythrocytes were detached from the buffy coat and plasma and washed three times with 10 volumes of 0.15 M NaCl. During the last washing, the erythrocytes were centrifuged at 2500 X*g* for 10 min to obtain a constantly packed cell preparation. Erythrocyte hemolysis was mediated by peroxyl radicals in this assay system^85^. A suspension (about 10%) of erythrocytes in pH 7.4 phosphate-buffered saline (PBS) was added to the same volume of 200 mM 2,2ʹ-azobis (2-amidinopropane) dihydrochloride (AAPH) solution (in PBS) containing samples of AgNPs to be tested. While the reaction mixture has incubated at 37 °C for approximately 1 h, it was shacked gently. The reaction mixture was then removed, diluted with eight volumes of PBS and centrifuged at 2500 X*g* for 10 min. The absorbance A (AgNPs) of the supernatant was read at 540 nm. Similarly, the reaction mixture was treated with eight volumes of distilled water to achieve complete hemolysis, and the absorbance B (dist. H_2_O) of the supernatant obtained after centrifugation was measured at 540 nm. The hemolysis percentage was calculated by equation (1−A/B) X100%. The data were displayed as mean standard deviation. Vitamin C (L- ascrobic) was used as a positive control.

#### *In vitro* cytotoxic effect of AgNPs on cancer and normal cell lines

The cytotoxic effect of AgNPs on Mammary gland breast cancer (MCF-7), human lung fibroblast (WI38) and human amnion (WISH) the cell lines which were obtained from ATCC via Holding company for biological products and vaccines (VACSERA), Cairo, Egypt was determined using *in vitro* colorimetric technique 3-(4, 5-Dimethyl thiazol-2yl)-2, 5-diphenyl tetrazolium bromide (MTT) assay which based on being split by NAD-dependent mitochondrial dehydrogenase of viable cells, resulting in purple color of the formazan product. This formazan production is directly proportional to the number of viable cell and inversely proportional to the cytotoxicity degree^86^. The cells were cultured in RPMI-1640 medium with 10% fetal bovine serum. Antibiotics added were 100 units/mL penicillin and 100 µg/mL streptomycin at 37 °C in a 5% CO_2_ incubator. The monolayer cells were detached with trypsin–ethylene diamine tetra acetic acid (EDTA) to make single cell suspensions. The viable cells were counted using a hemocytometer. The cell suspensions (100 µL/well) were seeded onto 96-well plates, maintaining the plating density as 10,000 cells/well, and then incubated for 48 h at 37 °C, 5% CO_2_, 95% air and 100% relative humidity for cell, attachment to the bottom of the wells. The cells were treated with serial concentrations of the AgNPs for 24 h. The AgNPs were previously passed through a 0.45 µm filter syringe. Serial dilutions were made to provide a total of seven samples concentrations. Various concentrations (1.56, 3.125, 6.52, 12.5, 25, 50, 100 µg/mL) of silver nanoparticles were inoculated into grown cell containing 100 µL medium. The plates were incubated for 24 h at 37 °C, 5% CO_2_, 95% air and100% relative humidity. Various concentrations (1.56, 3.125, 6.52, 12.5, 25, 50, 100 µM) of 5-fluorouracil (5-FU) were used as standard. The study was run in duplicate to ensure accuracy of the results. The yellow solution of 3-[4, 5-dimethylthiazol-2-yl] 2, 5- diphenyltetrazolium bromide (MTT) (20 µL) was added to phosphate buffered saline (5 mg/mL) in each well. The plates were incubated at 37 °C for 4 h for the reduction of MTT. The resulting purple formazan crystals were solubilized in 100 µL of DMSO and the colorimetric assay is measured and recorded at absorbance of 570 nm using a plate reader (EXL 800, USA). Cytotoxicity percentage was determined using the following equation:

4 $$\begin{array}{rcl}{\rm{Viability}}\, \%  & = & ({\rm{Test}}\,\text{OD}/\text{Control}\,{\rm{OD}})\times 100\\ {\rm{Cytotoxicity}}\, \%  & = & 100-{\rm{Viability}} \% \end{array}$$

#### *In vivo* cytotxicity of AgNPs on Ehrlich ascites tumor

Ethics statement. All experimental protocols were approved by Research Ethics Committee, Faculty of science, Mansoura University, Mansoura, Egypt. All the experiments were performed in accordance with the relevant guidelines and regulations.

Albino adult Swiss male mice (20–25 g) were obtained from Pharmacology Department, Mansoura University, Egypt and used throughout the study. They are housed in a controlled environment (temperature 25 ± 2 °C) and 12 hours dark/light cycle) with standard laboratory diet and water ‘*ad libitum*’. The Ehrlich ascites carcinoma (EAC) cells were provided by National Cancer Institute (NCI), Cairo, Egypt. EAC cells were diluted with normal saline (0.9% NaCl) to reach the desired concentration (2 × 10^6^ cells/0.2 mL).

The mice were divided into four groups comprising five animals in each group. Group I represents negative control - received vehicle (normal saline) with no EAC cells; group II served as tumor control injected with EAC (2 × 10^6^ cells per mouse); group III represents EAC bearing mice inoculated with 5-FU; group IV represents EAC bearing mice inoculated with AgNPs. So all groups were injected intraperitoneally with 2 × 10^6^ cells per mouse except group I. After 24 h, mice were injected with the anti cancer 5-FU and AgNPs (5 mg/kg body weight). The inoculation process was repeated for 10 days. After 24 h of the last dose including 18 h of fasting half of the mice in each group were weighed and sacrificed. Blood samples were collected from each group of sacrificed mice for the estimation of hemoglobin (Hb) content and counts for red (RBC) and white blood cells (WBC) were performed. Tumor cells volume and their count were determined by collecting the ascetic fluid from the peritoneal cavity of the mice. The count of viable cells and non-viable cells was estimated by taking a part of the ascetic fluid then centrifugated and stained with trypan blue (0.4% in normal saline) and the other part was centrifuged in a graduate centrifuge tube at 1,000 rpm for 5 min and the packed cell volume was measured. The increase of mice body weights were recorded both in the treated and control groups at the start of the experiment to the end (0 to 11 days).

## Conclusion

A biological method has been reported for the synthesis of silver nanoparticles using the proteinaceous pigment phycocyanin which is plentiful component of cyanobacterial cells. Phycocyanin extracted from *Nostoc linckia* was successfully used for biosynthesis of AgNPs. The face-centered central composite design has been used for the optimization of factors affecting AgNPs biosynthesis for attaining the maximum production of AgNPs. After statistical optimization by face-centered central composite design, the biosynthesis of silver nanoparticles by using phycocyanin extracted from *Nostoc linckia* was improved (1100.025 µg/mL) with fold of increase 2.6 as compared with that of unoptimized conditions (508.346 µg/mL). The physicochemical characterization viz. UV-Vis spectroscopy, zeta potential, FTIR, TEM and XRD confirmed the formation of AgNPs. The present study explores the potential antibacterial activity of AgNPs against Gram-positive and Gram-negative bacteria. The bio-AgNPs also displayed *in vitro* anticancer activity against mammary gland breast cancer (MCF-7) cell lines and showed low hemolysis activity (7.8%) of erythrocyte exposed to the free radicals of AAPH. Due to the enhanced antibacterial, anticancer and antihemolytic activity of AgNPs, it is effectively used in the field of medicine, besides pharmaceutical, food and cosmetic industries.

## Electronic supplementary material

Supplementary Information
