# Author Correction: Bio-fabrication of silver nanoparticles by phycocyanin, characterization, *in vitro* anticancer activity against breast cancer cell line and *in vivo* cytotxicity

**DOI:** 10.1038/s41598-022-06492-1

**Published:** 2022-02-07

**Authors:** Noura El-Ahmady El-Naggar, Mervat H. Hussein, Asmaa Atallah El-Sawah

**Affiliations:** 1 Department of Bioprocess Development, Genetic Engineering and Biotechnology Research Institute, City of Scientific Research and Technological Applications, Alexandria, Egypt; 2 Botany Department, Faculty of Science, Mansoura University, Mansoura, Egypt

Correction to: *Scientific Reports* 10.1038/s41598-017-11121-3, published online 7 September 2017

This Article contains an error in Figure 13, where the image of the cells treated with 5-FU (top left) is incorrect.

The correct Figure 13 and accompanying legend appear below.

**Figure 13:**
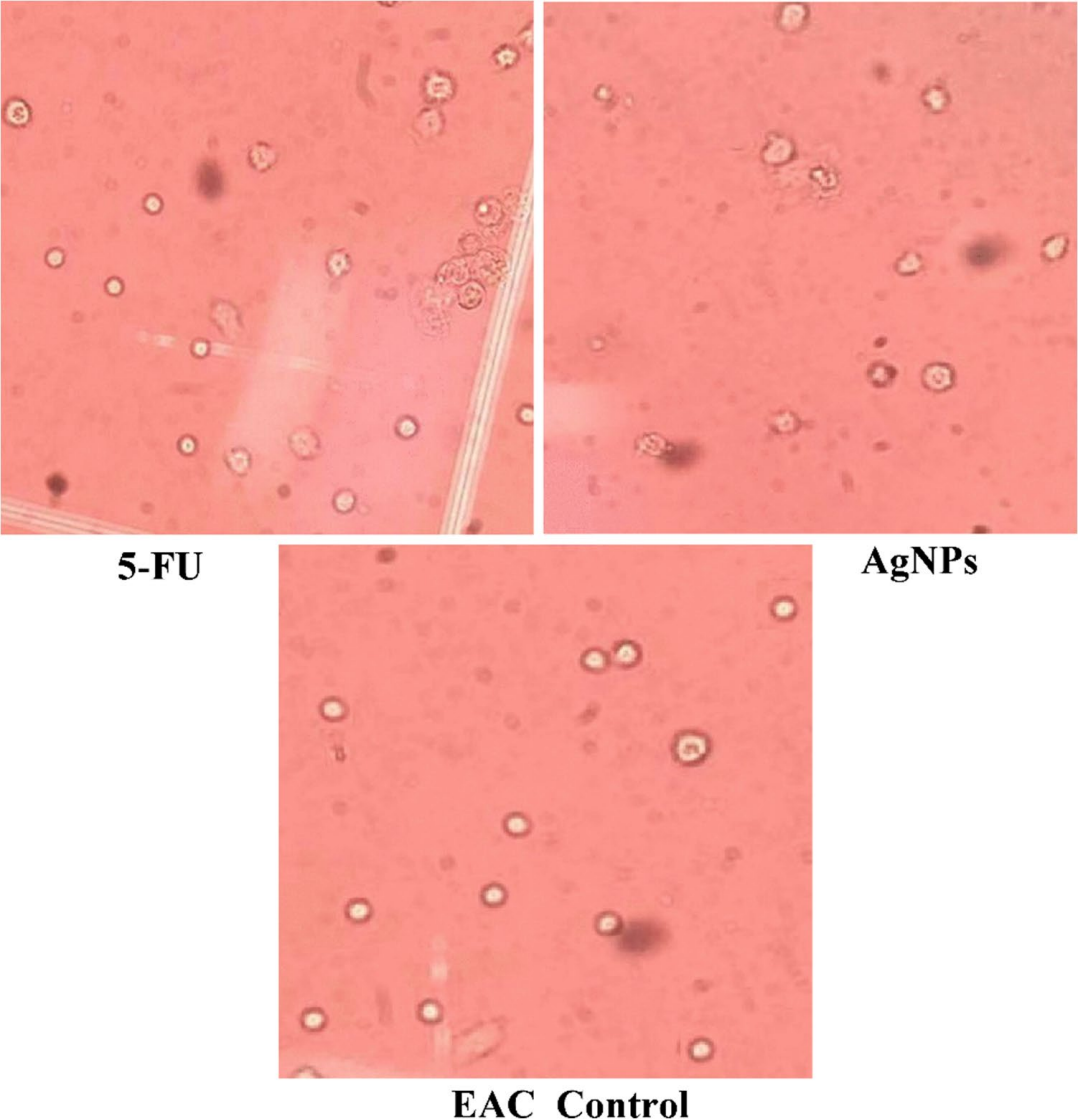
Microscopy images demonstrate the cytotoxic effect of AgNPs and 5-FU on EAC cells, cell damage by AgNPs due to loss of cell membrane integrity and apoptosis.

