# Supplementary material for: Bio-fabrication of silver nanoparticles by phycocyanin, characterization, in vitro anticancer activity against breast cancer cell line and in vivo cytotxicity
Source: Sci Rep. 2017 Sep 7;7:10844. doi: 10.1038/s41598-017-11121-3 (PMC5589729; doi:10.1038/s41598-017-11121-3)
Supplement: Supplementary file 1 — Supplementary Information [file 41598_2017_11121_MOESM1_ESM.pdf]

Bio-fabrication of silver nanoparticles by phycocyanin, characterization, *in vitro* anticancer activity against breast cancer cell line and *in vivo* cytotxicity

[1]Noura El-Ahmady El-Naggar*, [2]Mervat H. Hussein, [2]Asmaa Atallah El-Sawah

[1]Department of Bioprocess Development, Genetic Engineering and Biotechnology Research Institute, City of Scientific Research and Technological Applications, Alexandria, Egypt

[2]Botany Department, Faculty of Science, Mansoura University, Mansoura - Egypt

Running title: Bio-fabrication of silver nanoparticles by phycocyanin

*To whom correspondence should be addressed.

Dr. Noura El-Ahmady Ali El-Naggar

Address:

Bioprocess Development Department,

Genetic Engineering and Biotechnology Research Institute,

City of Scientific Research and Technological Applications,

New Borg El- Arab City, 21934, Alexandria, Egypt

Tel: (002)01003738444

Fax: (002)03 4593423

E-mail: nouraelahmady@yahoo.com

The fit summary results are presented in Supplementary Table S1. The aim of sequential model sum of squares is to select the highest order polynomial where terms are significant; quadratic model type was selected to be the proper model that fit the FCCD of silver nanoparticles biosynthesis by phycocyanin pigment extracted from *Nostoc linckia*, where fit summary results showed that, the quadratic model is a highly significant model with a very low probability value [($P_{model} > F$) < 0.0001]. The model summary statistics focus on the models that have lower standard deviation and higher adjusted and predicted R-squared; the model summary statistics of the quadratic model showed the smallest standard deviation of 71.18 and the largest adjusted and predicted R-squared of 0.9489 and 0.8250 respectively.

Supplementary Table S1. Fit summary for face-centered central composite design experimental values of silver nanoparticles biosynthesis by phycocyanin pigment.

**Sequential Model Sum of Squares**					
**Source**	**Sum of Squares**	***Df***	**Mean Square**	***F*-value**	***P*-value Prob >*F***
Linear vs Mean	111143.48	4	27785.87	0.25	0.9061
Two factors interaction vs Linear	49123.49	6	8187.25	0.06	0.9990
Quadratic vs two factors interaction	2637799.81	4	659449.95	130.16	< 0.0001*
Residual	34628.38	7	4946.91		
**Lack of Fit Tests**					
**Source**	**Sum of Squares**	***Df***	**Mean Square**	***F*-value**	***P*-value Prob >*F***
Linear	2762901.46	20	138145.07	41614.18	< 0.0001*
Two factors interaction	2713777.97	14	193841.28	58391.85	< 0.0001*
Quadratic	75978.15	10	7597.82	2288.73	< 0.0001*
Pure Error	16.60	5	3.32		
**Model Summary Statistics**					
**Source**	**Standard deviation**	**R-Squared**	**Adjusted R-Squared**	**Predicted R-Squared**	**PRESS**
Linear	332.44	0.0387	-0.1151	-0.3281	3817156.96
Two factors interaction	377.93	0.0558	-0.4412	-2.1953	9183470.43
Quadratic	71.18	0.9736	0.9489	0.8250	503061.47

* Significant values, *df* : degree of freedom, PRESS: sum of squares of prediction error
